# Revealing the efficacy-toxicity relationship of Fuzi in treating rheumatoid arthritis by systems pharmacology

**DOI:** 10.1038/s41598-021-02167-5

**Published:** 2021-11-29

**Authors:** Wuwen Feng, Juan Liu, Dandan Zhang, Yuzhu Tan, Hao Cheng, Cheng Peng

**Affiliations:** 1 State Key Laboratory of Southwestern Chinese Medicine Resources, School of Pharmacy, Chengdu University of Traditional Chinese Medicine, Chengdu, 611130 China; 2 Key Laboratory of the Ministry of Education for Standardization of Chinese Medicine, Chengdu University of Traditional Chinese Medicine, Chengdu, 611130 China

**Keywords:** Systems analysis, Data publication and archiving, Virtual drug screening

## Abstract

In recent decades, herbal medicines have played more and more important roles in the healthcare system in the world because of the good efficacy. However, with the increasing use of herbal medicines, the toxicity induced by herbal medicines has become a global issue. Therefore, it is needed to investigate the mechanism behind the efficacy and toxicity of herbal medicines. In this study, using *Aconiti Lateralis* Radix Praeparata (Fuzi) as an example, we adopted a systems pharmacology approach to investigate the mechanism of Fuzi in treating rheumatoid arthritis and in inducing cardiac toxicity and neurotoxicity. The results showed that Fuzi has 25 bioactive compounds that act holistically on 61 targets and 27 pathways to treat rheumatoid arthritis, and modulation of inflammation state is one of the main mechanisms of Fuzi. In addition, the toxicity of Fuzi is linked to 32 compounds that act on 187 targets and 4 pathways, and the targets and pathways can directly modulate the flow of Na^+^, Ca^2+^, and K^+^. We also found out that non-toxic compounds such as myristic acid can act on targets of toxic compounds and therefore may influence the toxicity. The results not only reveal the efficacy and toxicity mechanism of Fuzi, but also add new concept for understanding the toxicity of herbal medicines, i.e., the compounds that are not directly toxic may influence the toxicity as well.

## Introduction

With the usage history over hundreds of or even thousands of years, many complementary and alternative medicines harbor rich clinical experiences. Because of the high safety and rich clinical experiences, the use of complementary and alternative medicines such as traditional Chinese medicines has increased globally over the past decades. Herbal medicines are an important resource of complementary and alternative medicines, and they play important roles in the current medical system. Reports published by the World Health Organization (WHO) showed that about 75–80% of the world population, especially those in developing countries, rely primarily on herbal medicines for healthcare^1^. In addition, compounds isolated from herbal medicines are important resources of lead compounds in drug discovery area. For example, it is reported that 63% of anticancer small molecular drugs approved by the US Food and Drug Administration (FDA) are directly or indirectly come from herbal medicines^2^. Recently, herbal medicines have played significant roles in fighting against COVID-19 before the wide application of vaccines^3,4^. Predictably, herbal medicines will play more and more important roles in fighting against diseases.

Even though herbal medicines have been demonstrated to be effective on many diseases, the toxicity caused by herbal medicines has become a global issue. For example, aristolochic acids are a group of herbal compounds that widely exist in the plant genus *Aristolochia* and *Asarum*. Since the 1990s, aristolochic acids-induced nephropathy and upper tract urothelial carcinoma have been reported in countries such as Belgium, UK, France, Japan, and China^5^. In 2012, the WHO cancer agency the International Agency for Research on Cancer (IARC) classified aristolochic acids as group 1 human carcinogens^6^. Gradually, the use of aristolochic acids-containing herbal medicines is forbidden in many countries. Except for the herbal medicines that contain aristolochic acids, there are many herbal medicines with strong toxicity that are still used in the clinic. This is because the toxicity of these herbal medicines can be eliminated with careful use. However, the reason an herbal medicine can lead to both efficacy and toxicity is not fully known.

Among all herbal medicines, *Aconiti Lateralis* Radix Praeparata (Fuzi) is the typical herbal medicine with conspicuous efficacy and strong toxicity. Fuzi, also known as Chinese wolfsbane, Chinese aconite, monkshood, Kyeong-Po Buja, and Bushi, is the processed daughter root of *Aconitum carmichaeli* Debx. As one of the most well-known herbal medicines, Fuzi has been extensively used for over 2 thousand years in clinics to treat diseases^7^. In the clinic, Fuzi is commonly used to treat a wide variety of diseases, such as rheumatoid arthritis, acute myocardial infarction, low blood pressure, coronary heart disease, chronic heart failure, tumors, skin wounds, depression, diarrhea, gastroenteritis, and edema^8^. Although Fuzi has shown wide and promising therapeutic effects, its toxicity has been recognized by ancient people and has attracted widespread attention all over the world in the past decades. From 2001 to 2010, 5000 cases of acute toxicity of Fuzi were recorded in countries such as China, Japan, and Germany^9^. From 2004 to 2015 in mainland China, at least 40 cases of fatal Fuzi poisoning with 53 victims were recorded^10^. The main toxicity of Fuzi includes cardiotoxicity and neurotoxicity, and the typical symptoms of Fuzi poisoning include arrhythmia, palpitation, hypotension, shock, dizziness, coma, vomiting, and nausea^11^. Although the potent therapeutic effects and strong toxicity of Fuzi are well recognized, it is now still not fully understood that which compounds are responsible for the efficacy and toxicity, and the mechanism differences of Fuzi in inducing efficacy and toxicity.

**Figure 1:**
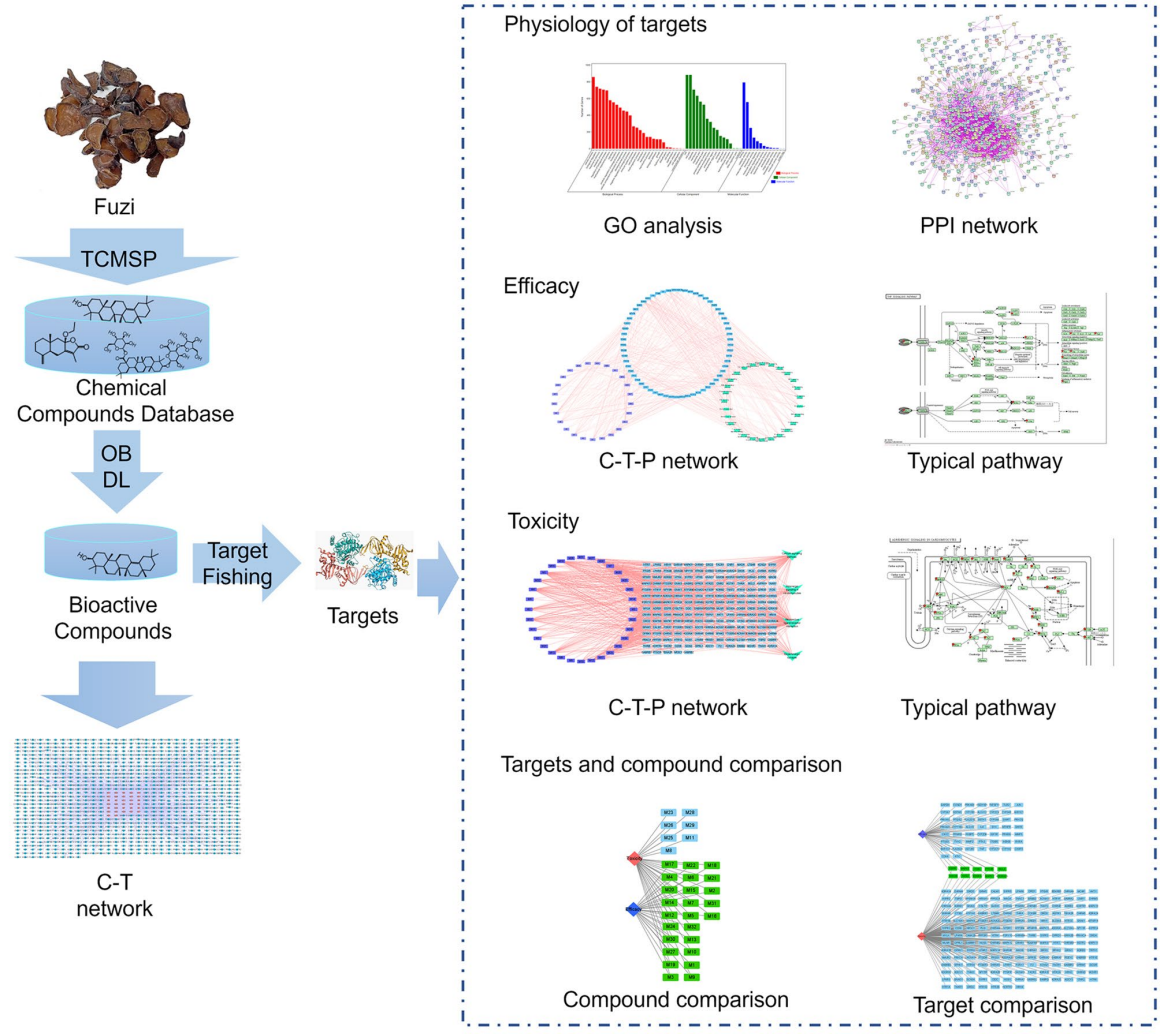
Schematic diagram of the systems pharmacology to comparatively investigate the efficacy and toxicity mechanisms of Fuzi. *C-T* compound-target, *C-T-P* compound-target-pathway, *DL* drug-likeness, *Fuzi* *Aconiti Lateralis* Radix Praeparata, *GO* gene ontology, *OB* oral bioavailability, *PPI* protein–protein interaction, *TCMSP* Traditional Chinese Medicine Systems Pharmacology Database and Analysis Platform.

Different from synthesized chemical drugs that only contain one or two chemical compounds, an herbal medicine usually contains hundreds or even thousands of different compounds that act holistically to treat diseases^12^. The traditional wet experiment-based approach is rather difficult to identify all the bioactive compounds and their mechanisms. Fortunately, with the development of systems biology, systems pharmacology has emerged as an efficient tool to study the mechanism of herbal medicines based on the complex status of the biological system of the human body^13^. For this reason, it has been extensively and successfully used to study the mechanisms of herbal medicine such as *Morinda officinalis*^14^. In this work, we adopted a standard systems pharmacology approach to screen the compounds that are responsible for the efficacy and toxicity of Fuzi in treating arthritis and inducing toxicity, respectively (Fig. 1). Meanwhile, the mechanisms of efficacy and toxicity were investigated and the mechanism differences between efficacy and toxicity were compared. This work can be helpful for a comprehensive understanding of the efficacy-toxicity relationship of toxic herbal medicines in treating diseases.

## Methods

### Chemical compounds retrieving and active compounds screening of Fuzi

All the compounds and molecular structures of Fuzi were retrieved and downloaded from Traditional Chinese Medicine Systems Pharmacology Database and Analysis Platform (TCMSP, http://ibts.hkbu.edu.hk/LSP/tcmsp.php), a systems pharmacology platform that is specialized for traditional Chinese medicines^15^. Considering that gut microbiota can convert glycosides in the intestinal tract by gut microbiota^16^, the corresponding aglycones were also included in the compound library of Fuzi. To screen the potential bioactive compounds from Fuzi, two important parameters including oral bioavailability (OB) and drug-likeness (DL) that are associated with drug absorption, distribution, metabolism, and excretion were used. The threshold for screening bioactive compounds was set as OB ≥ 30 and DL ≥ 0.18^17^. Because some compounds that did not pass the threshold might show significant pharmacological effects as well, compounds that were reported to exhibit strong pharmacological effects yet not meet the threshold of OB or DL were also used.

### Target screening

To screen the whole targets of bioactive compounds in Fuzi, a chemometric method and information integration approach were used. First, the screened bioactive compounds were submitted to various on-line servers and databases, including Bioinformatics Analysis Tool for Molecular Mechanism of Traditional Chinese Medicine (BATMAN-TCM, http://bionet.ncpsb.org/batman-tcm/index.php/Home/Index/index)^18^. Similarity Ensemble Approach (SEA, http://sea.bkslab.org)^19^, TCMSP (http://ibts.hkbu.edu.hk/LSP/tcmsp.php) ^15^, Therapeutic Targets Database (TTD, http://bidd.nus.edu.sg/group/ttd/) ^20^, PhID (http://phid.ditad.org/MetaNet/)^21^, and Swiss Target Prediction (STP, http://www.swisstargetprediction.ch/) ^22^. It is noteworthy that only the targets related to *Homo sapiens* were reserved for further analysis. P-value less than 0.05 were used for screening of targets in on-line serves and database where applicable. The other parameters for target screening were defaultly settled, for example, Score cutoff with 20 was used for BATMAN-TCM.

### Targets validation

Molecular docking was performed using crystal structure of ADRA2A, BCHE, CHRM2, KCNH2 from Protein Data Bank (corresponds to PDB ID: 6KUX, 4BDS, 5ZKC, 5VA2, respectively). Compound structures of coryneine, denudatine, norcoclaurine, songorine were downloaded from TCMSP. Compound structures were performed energy minimization calculation by Chem 3D software and then imported into AutoDockTools for adding hydrogen and computing gasteiger. Crystal structures were separated from original ligands and were prepared by AutoDockTools through removing water, adding hydrogen and computing gasteiger. The virtual docking was implemented in the AutoDock Vina. The best docking pose were predicated based on the docked free energy and inhibition constant. The 3D binding model was shown by Pymol, 2D shown by LigPlus.

### Gene ontology and protein–protein interaction analysis

To gain the biological, molecular, and cellular function of target genes, gene ontology (GO) analysis including biological process (BP), cellular component (CC), and molecular function (MF) was performed. An on-line web tool OmicShare was used to carry GO analysis (https://www.omicshare.com/tools/). The protein–protein interaction (PPI) network of targets acquired from database STRING (https://string-db.org/, version 11.0). For visual of the PPI network, the line color indicated the type of interaction evidence, the active interaction sources were based on experiments, and the disconnected nodes were excluded in the final network.

### Screening of efficacy and toxicity-related targets and mechanisms

The genes and proteins related to rheumatoid arthritis were retrieved from Comparative Toxicogenomics Database (CTD, http://ctdbase.org/ )^23^ and DrugBank (https://go.drugbank.com/) to obtain the efficacy-related targets^24^, and only those genes with direct evidence to rheumatoid arthritis were reserved. The efficacy-related targets of Fuzi are those genes that belong to the targets of Fuzi and are directly linked to rheumatoid arthritis. To obtain the mechanism of Fuzi in treating rheumatoid arthritis, the efficacy-related targets of Fuzi were first subjected to Webserver the Database for Annotation, Visualization and Integrated Discovery (DAVID, https://david.ncifcrf.gov/home.jsp), and only the no disease pathways with *P* < 0.05 were reserved^25^. To obtain the targets and mechanisms of the toxicity of Fuzi, all the target genes were subjected to DAVID. Because the main toxicity of Fuzi is cardiac toxicity and neurotoxicity, and ion channels are involved in the onset of toxicity^26^, the enriched pathways including calcium signaling, adrenergic signaling in cardiomyocytes, neuroactive ligand-receptor interaction, dopaminergic synapse were considered as the toxic pathways of Fuzi, and all the target genes on those pathways were reserved.

### Network construction

To visualize the association among the bioactive compounds, disease-related potential targets, and toxicity-related targets and pathways, 3 networks were constructed including compound-target network for all active compounds and targets of Fuzi, compound-target-pathway network for treatment of rheumatoid arthritis by Fuzi, compound-target-pathway network for toxicity of Fuzi. To visualize the relationship compound and mechanism difference between efficacy and toxicity, the efficacy/toxicity-compound and the efficacy/toxicity-target networks were constructed. Cytoscape (version 3.8.2) was used for the visualization of networks.

## Results

### Bioactive compounds and putative targets of Fuzi

After screening of bioactive compounds by OB and DL, a total of 22 compounds were screened. In addition, the compounds that did not meet the criteria of OB and DL but were reported to show pharmacological effects were also preserved. These compounds include myristic acid, mesaconitine, etc. As a result, a total of 32 bioactive compounds were screened (Table 1). These compounds mainly belong to alkaloids. Target fishing showed that these compounds can act on 905 targets, and all the targets were shown in Supplementary Table S1. To help view the relationship between bioactive compounds and the corresponding targets, a compound-target network was constructed (Supplementary Fig. S1).

**Table 1:**
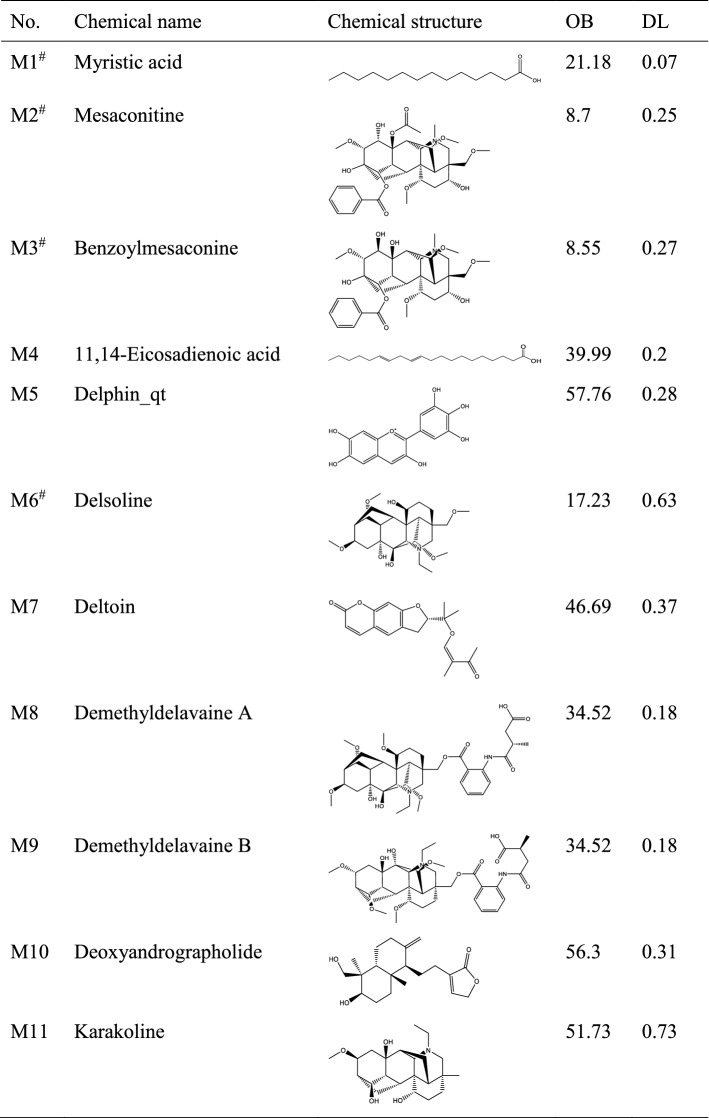

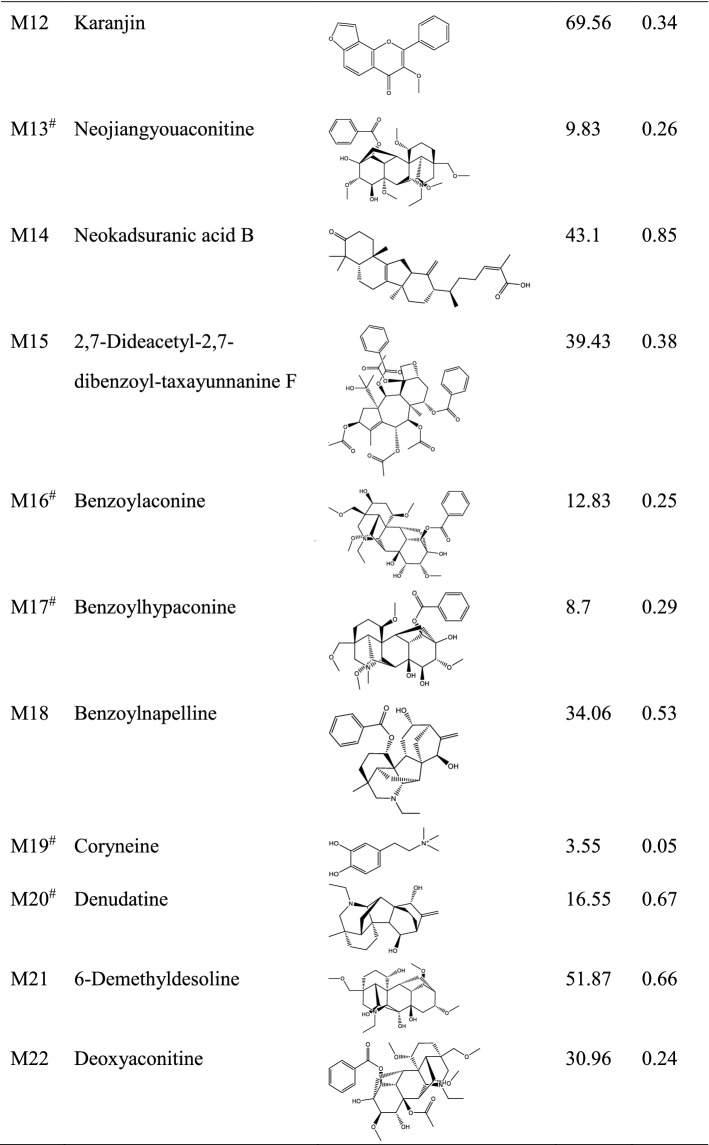

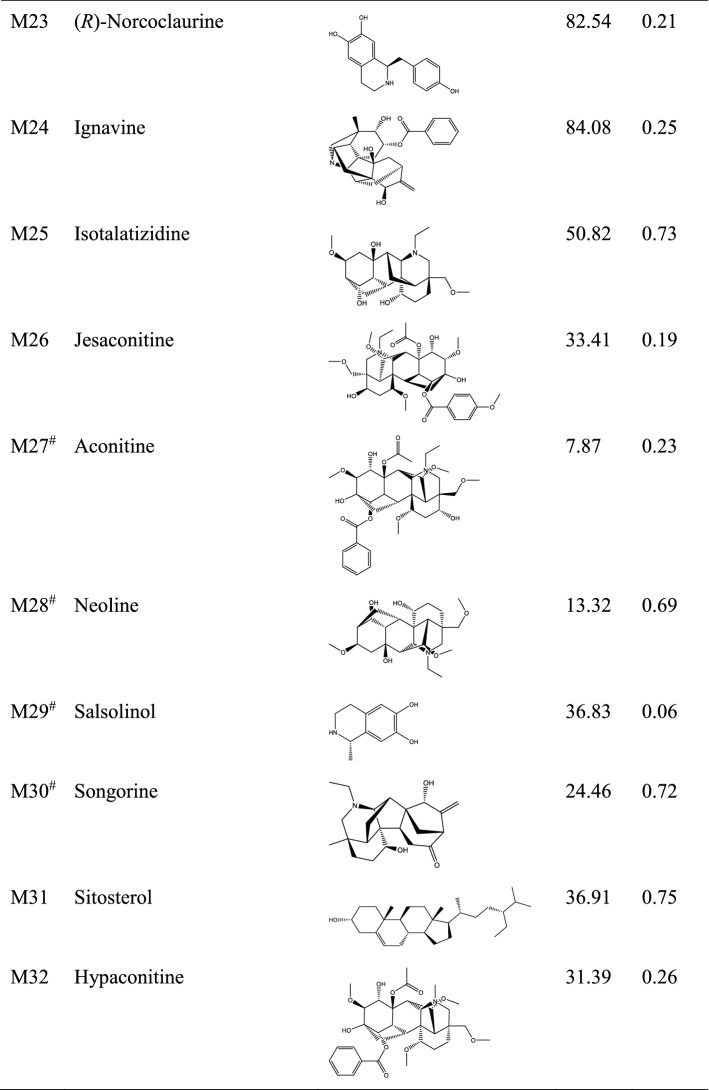
Bioactive compounds and key parameters of Fuzi.

*OB* oral bioavailability, *DL* drug-likeness.

^#^Compounds with OB < 30% and/or DL < 0.18, yet pharmaceutically validated.

### Molecular docking

**Figure 2:**
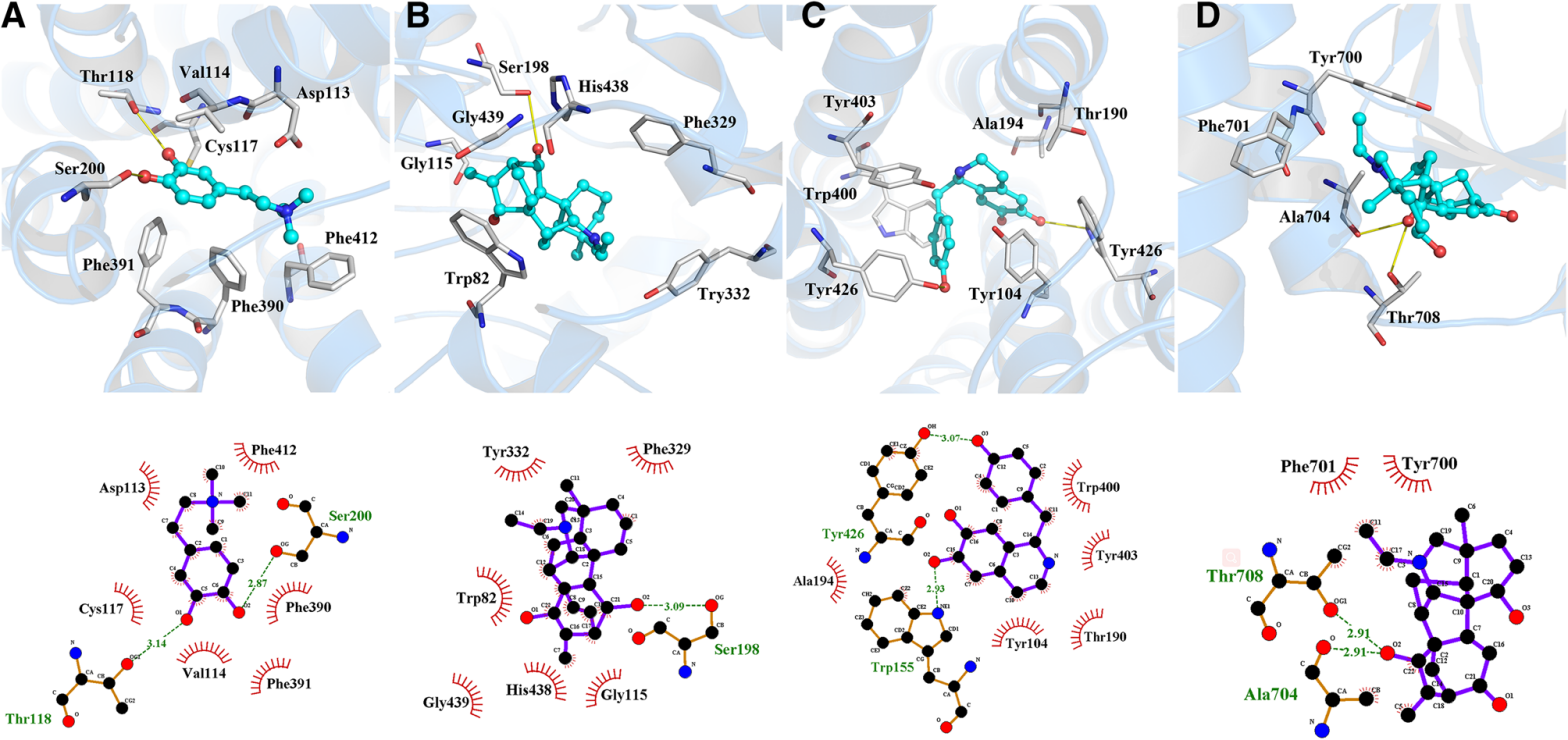
Molecular docking simulation for bioactive ingredients and target proteins. **(A)** Coryneine and ADRA2A, **(B)** denudatine and BCHE, **(C)** norcoclaurine and CHRM2, **(D)** songorine and KCNH2.

To verify the reliability of targets screened, molecular docking was utilized to estimate the binding ability between compounds of Fuzi and targets, and to view the compound-target binding interactions. The bioactive ingredients in Fuzi exhibited a strong binding ability towards the predicted genes (Fig. 2). The results showed that coryneine can bind to amino acid residue Thr118 and Ser200 in ADRA2A by hydrogen bonds (Fig. 2A). Denudatine can bind to amino acid residue Ser198 in BCHE by hydrogen bonds (Fig. 2B). Norcoclaurine can bind to amino acid residue Trp155 and Tyr426 in CHRM2 by hydrogen bonds (Fig. 2C). Songorine can bind to amino acid residue Thr708 and Ala704 in KCNH2 by hydrogen bonds (Fig. 2D). The binding energy of these four binding interactions were − 6.4, − 9.5, − 9.0, − 6.6 kcal/mol, respectively, indicating the reliability of targets in our study.

### PPI network construction and GO analysis

**Figure 3:**
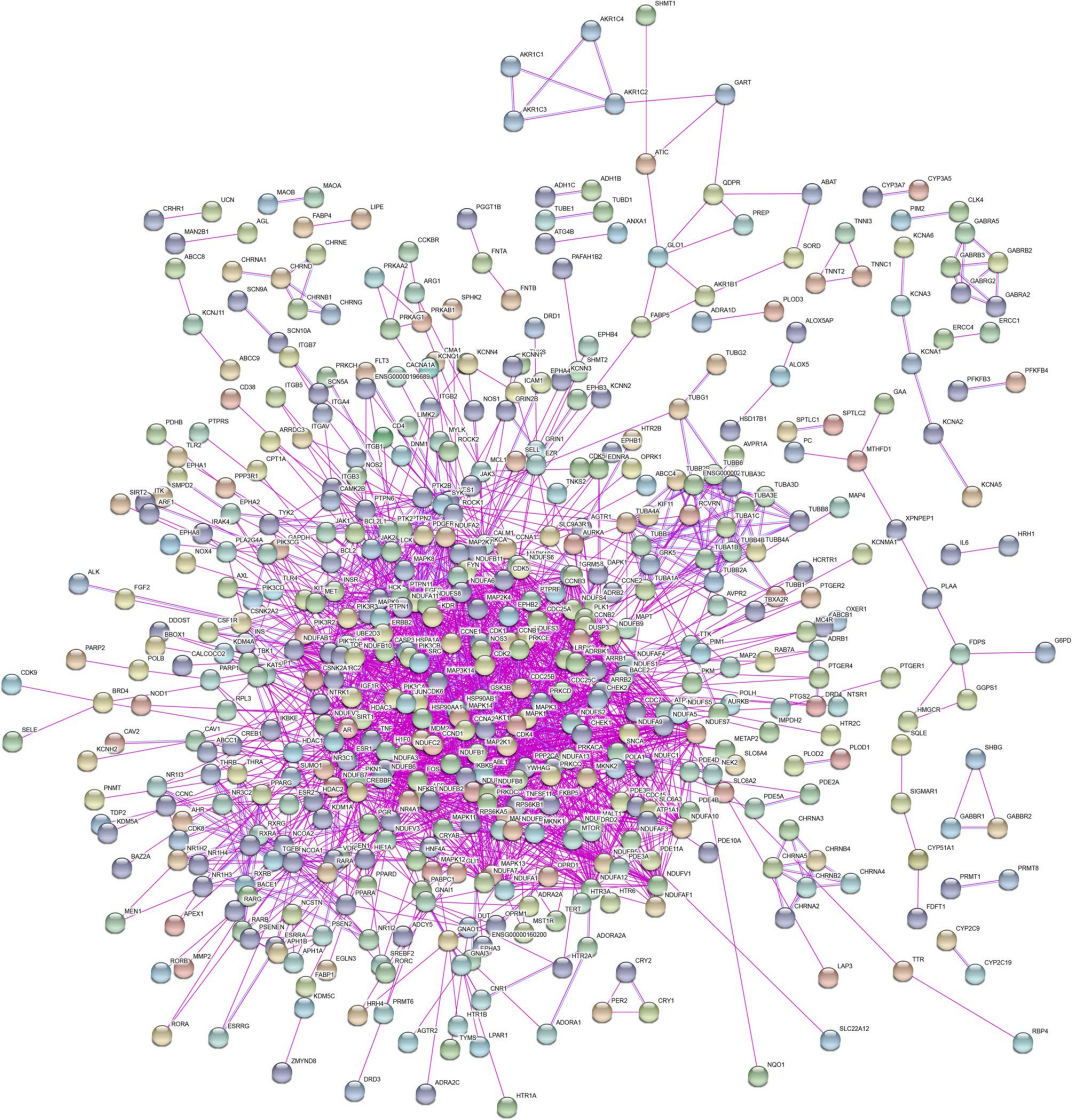
Protein–protein interaction network showing the relationships among targets of Fuzi. The nodes without linkage with other nodes were excluded from this network.

**Figure 4:**
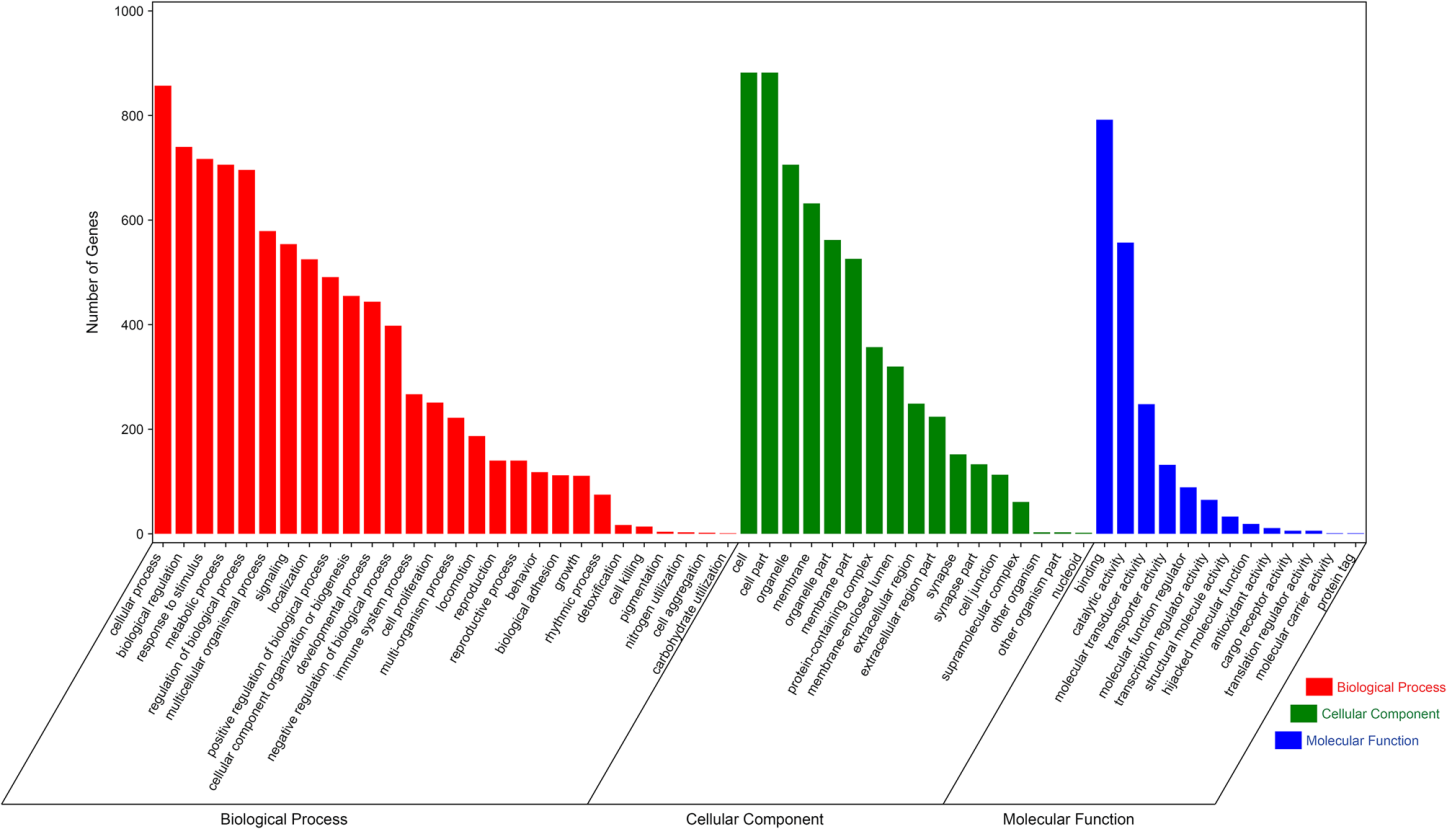
Gene ontology enrichment analysis showing the number of targets participating in biological process, cellular component, and molecular function.

To visualize the properties of targets, first, a PPI network was constructed with the help of String database. Results showed that a total of 902 targets were integrated into the whole PPI network. The number of edges was 2095, and the average node degree was 4.64 (Fig. 3). Noteworthy is that the PPI network contained several small networks. For example, three targets including SHBG, GABBR1, and GABBR2 were integrated as a small network. Then, GO analysis was performed with the help of Omicshare (Fig. 4). The results showed that the top 5 biological processes include cellular process, biological regulation, response to stimulus, metabolic process, and regulation of biological process. The 5 top cellular components include cell, cell part, organelle, membrane, and organelle part. The 5 top molecular functions include binding, catalytic activity, molecular transducer activity, transporter activity, and molecular function regulator.

### Efficacy mechanism of Fuzi

**Figure 5:**
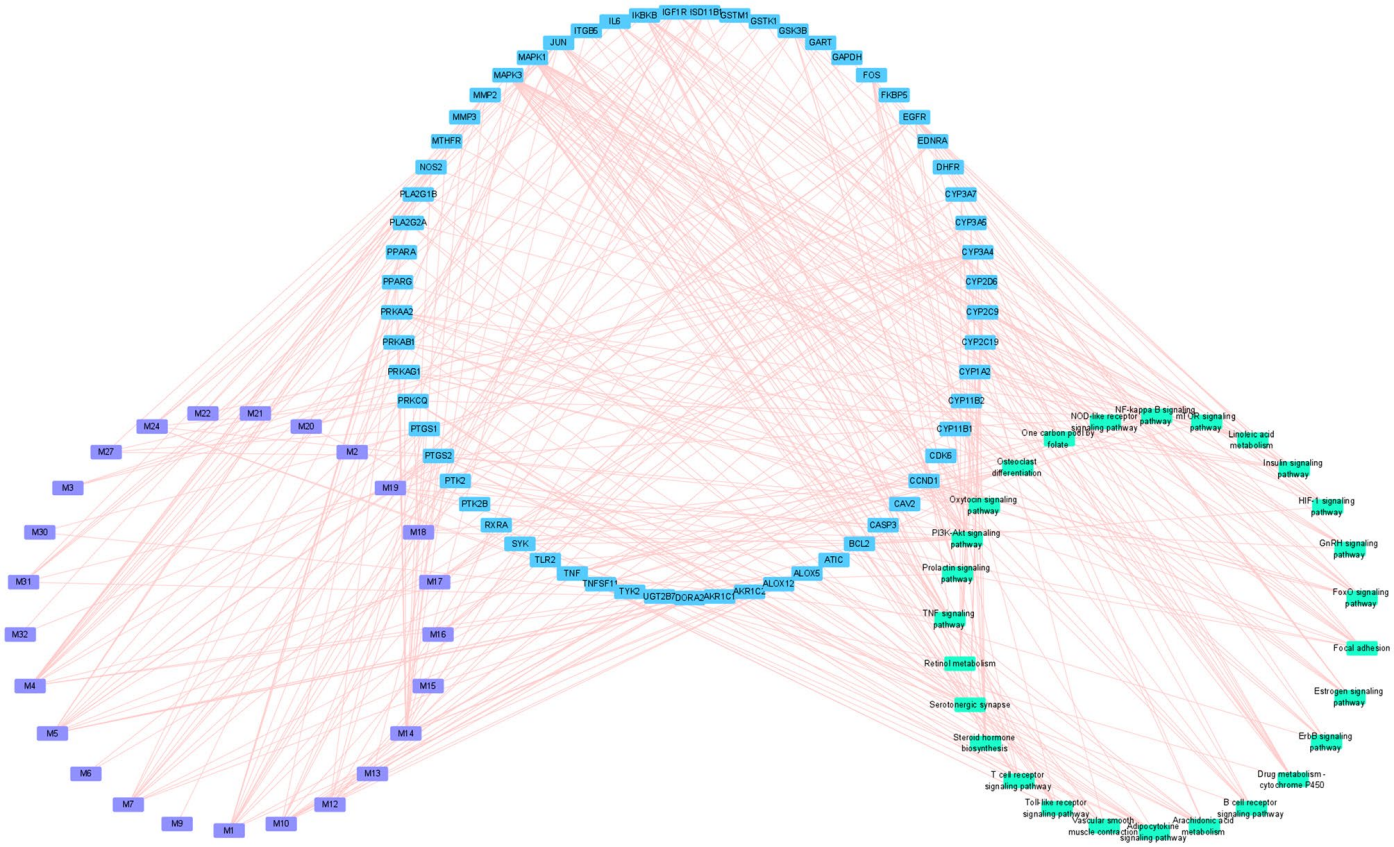
Compound-target-pathway network showing the comprehensive mechanism of Fuzi in treating rheumatoid arthritis. From left to right, the nodes in three big circles correspond to compounds in Fuzi, targets of Fuzi responsible for the treatment of rheumatoid arthritis, and the enriched non-disease pathways of the targets. The full name of compounds and targets are shown in Table 1 and Supplementary Table S1, respectively.

**Figure 6:**
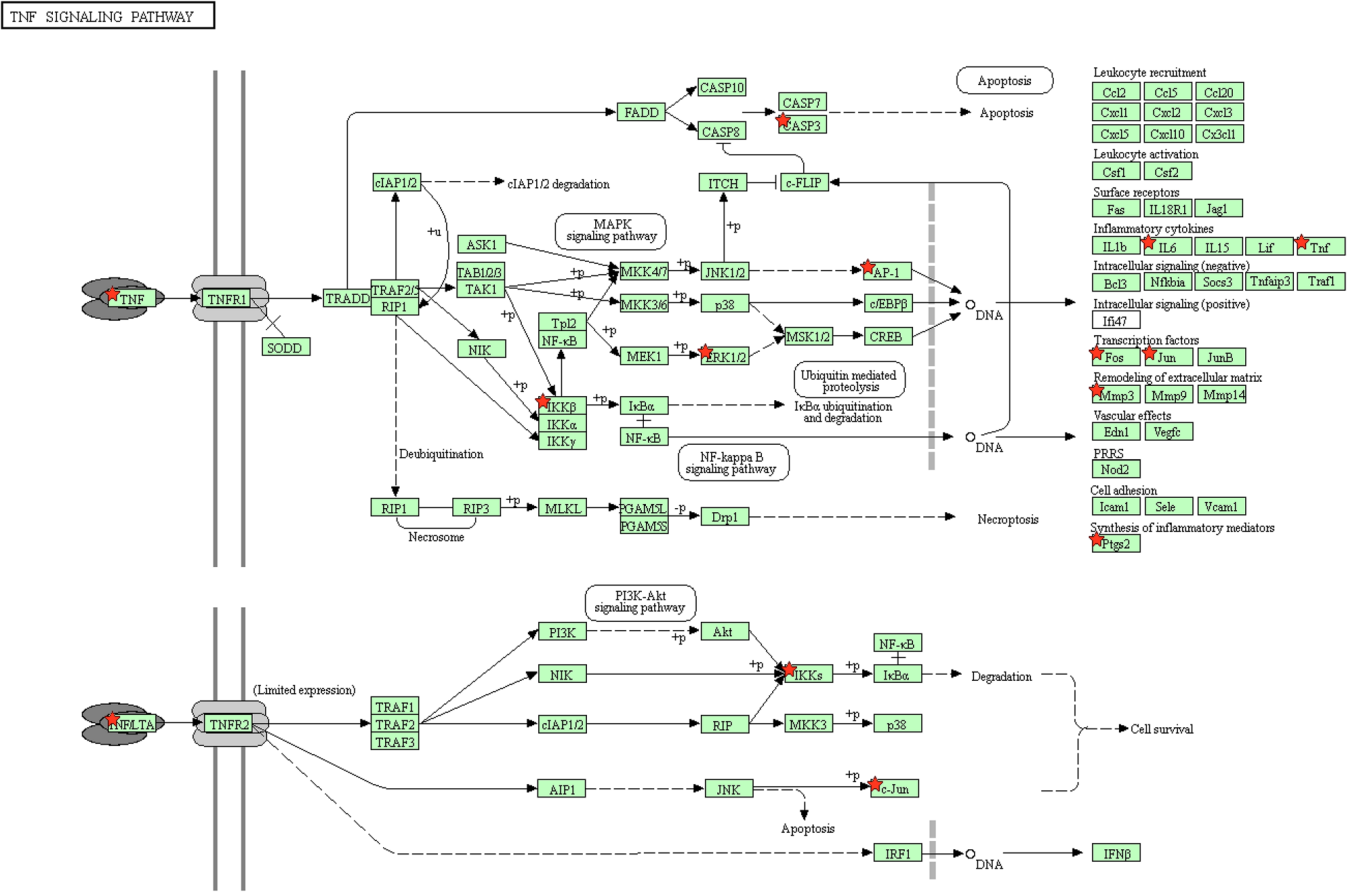
Distribution of the targets of Fuzi on the compressed TNF signaling pathway. The nodes with red star correspond to the targets of Fuzi, and the light blue nodes are targets in TNF signaling pathway. The compressed pathway was obtained from KEGG.

To explore the mechanism of Fuzi in treating rheumatoid arthritis, the targets were further screened by DrugBank and CTD. The screened targets associated with efficacy were further subjected to DAVID pathway analysis. As a result, a total of 27 pathways were enriched. These pathways include TNF signaling pathway, serotonergic synapse, arachidonic acid metabolism, adipocytokine signaling pathway, linoleic acid metabolism, PI3K-Akt signaling pathway, steroid hormone biosynthesis, etc. Many of these pathways have been demonstrated to be involved in rheumatoid arthritis. For example, modulation of arachidonic acid metabolism and linoleic acid metabolism is believed as two possible ways to treat arthritis^27,28^. To help visualize the mechanism of Fuzi, a compound-target-pathway network was constructed (Fig. 5). This network contains 25 bioactive compounds, 61 targets, and 27 pathways. It is noteworthy that in this network, one compound can act on multiple targets, and each pathway contains multiple targets. For example, myristic acid (M1) can act on TLR2, UGT2B7, RXRA, PTGS1, PTGS2, PPARG, PPARA, PLA2G1B, PLA2G1A, HSD11B1, GSTM1, GSTK1, EDNRA, and ALOX12. TNF pathway plays important roles in the development of rheumatoid arthritis and can be used as a target pathway for the treatment of rheumatoid arthritis^29^. Fuzi can act on multiple targets in the TNF pathway (Fig. 6). These results showed that Fuzi could exert therapeutic effects by influencing multiple targets and pathways, featuring the property that herbal medicine act in a holistic way to treat disease.

### Toxicity mechanism of Fuzi

**Figure 7:**
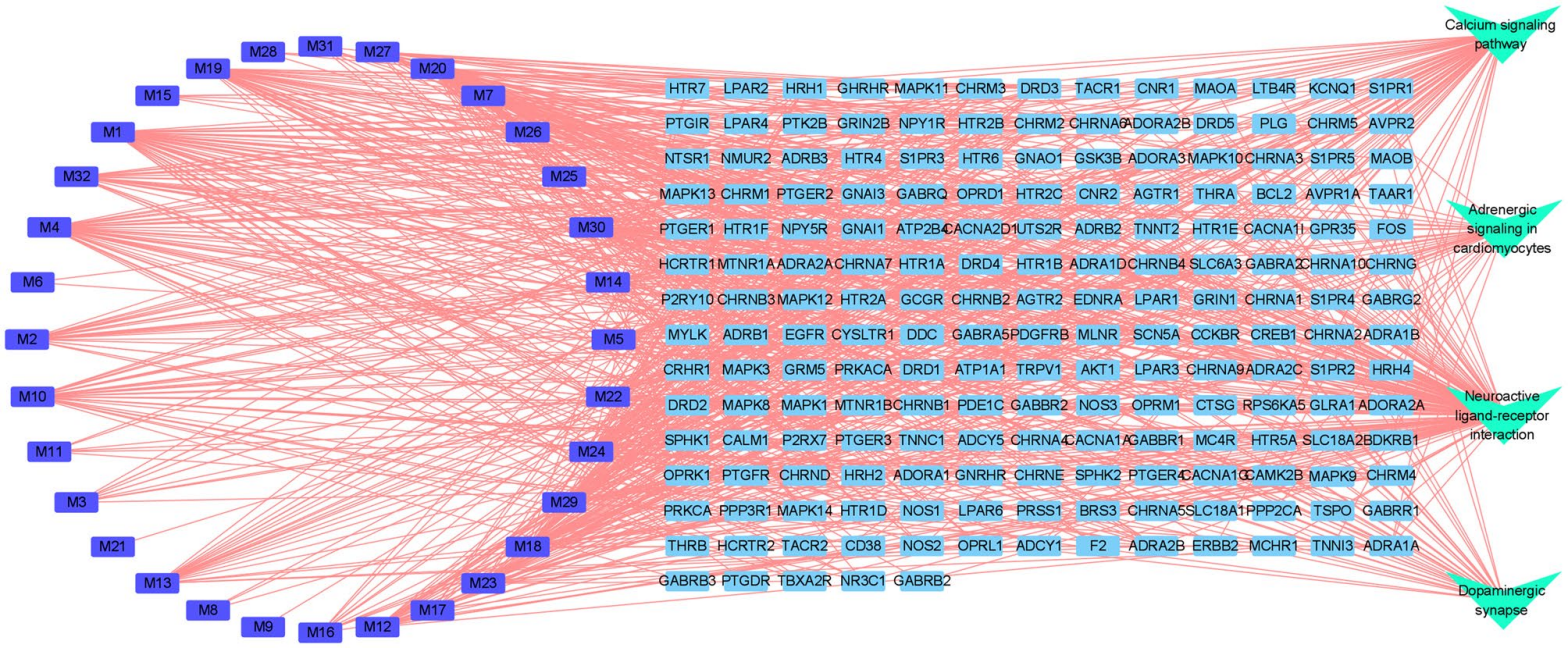
Compound-target-pathway network showing the comprehensive toxic mechanism of Fuzi. From left to right, the nodes correspond to compounds in Fuzi, targets of Fuzi responsible for toxicity, and the enriched pathways responsible for the toxicity. The full name of compounds and targets are shown in Table 1 and Supplementary Table S1, respectively.

**Figure 8:**
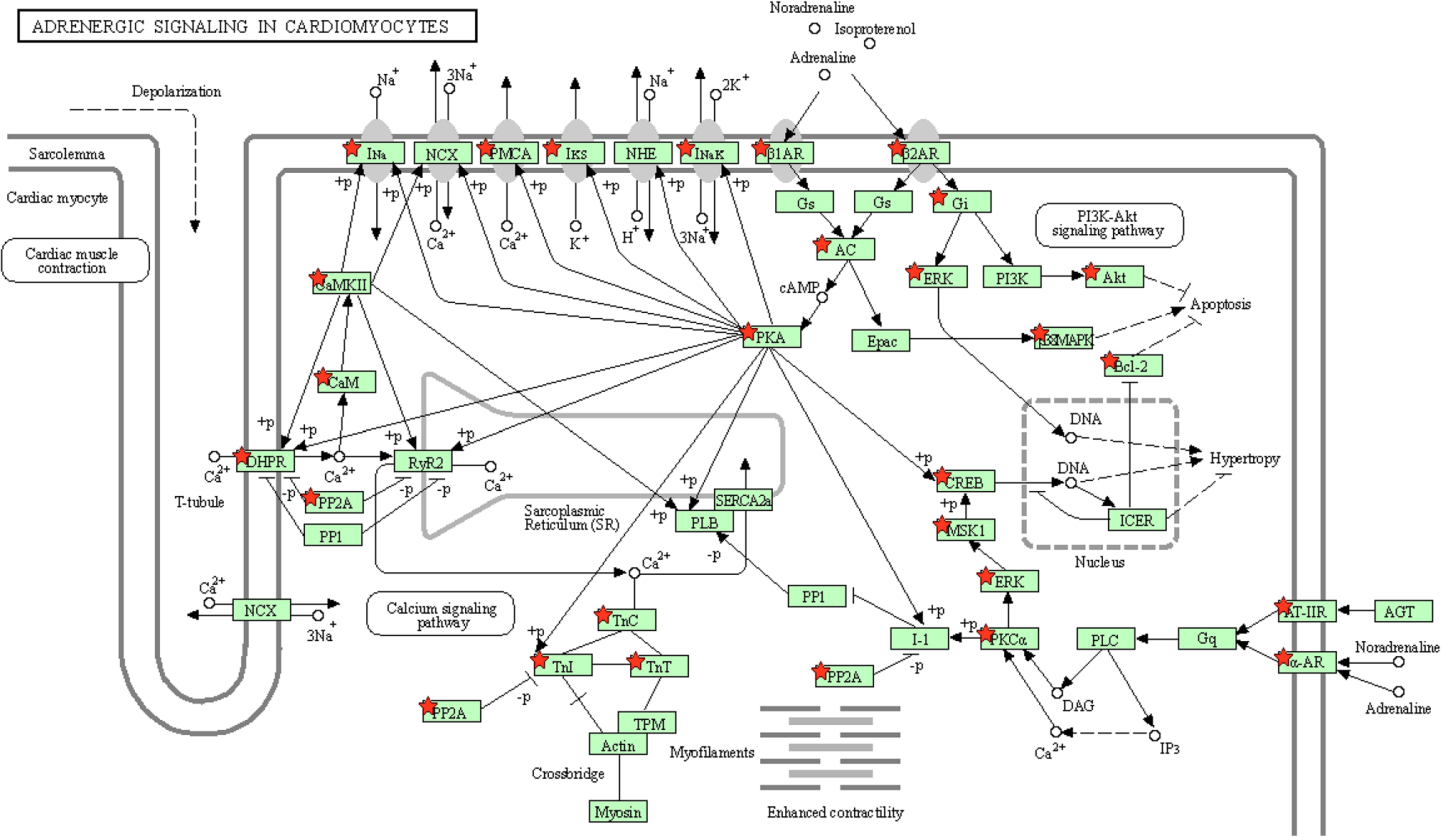
Distribution of the targets of Fuzi on the compressed adrenergic signaling in cardiomyocytes pathway. The nodes with red stars correspond to the targets of Fuzi, and the light blue nodes are targets in TNF signaling pathway. The compressed pathway was obtained from KEGG.

To fully explore the toxic mechanism of Fuzi, all the targets of Fuzi were subjected to KEGG analysis. Because the main toxicity of Fuzi includes cardiotoxicity and neurotoxicity, and disturbance of ion channels such as voltage-gated Na^+^ channel is responsible for toxicity^26^, therefore calcium signaling, adrenergic signaling in cardiomyocytes, neuroactive ligand-receptor interaction, and dopaminergic synapse were considered as the toxic pathways of Fuzi. To help visualize the comprehensive toxic mechanism of Fuzi, a compound-target-pathway network was constructed (Fig. 7). In this network, 32 compounds can act on 187 targets, and these targets can act on 4 pathways. It should be noted that in this network, one compound can act on multiple targets, and each pathway contains multiple targets. For example, hypaconitine (M23) can act on TBXA2R, SLC6A3, OPRM1, OPRL1, OPRK1, OPRD1, MTNR1B, MLNR, MAOA, HTR1A, HCRTR2, HCRTR1, DRD5, DRD4, DRD3, DRD2, DRD1, CHRNB4, CHRNA3, CHRM4, ADRB3, ADRB2, ADRA2C, ADRA2A, ADRA1D, ADRA1B, ADRA1A, and ADCY5. Similarly, multiple targets can act on the same pathway such as adrenergic signaling in cardiomyocytes (Fig. 8). Noteworthy is that disturbance of ion flow including Na^+^, Ca^2+^, and K^+^ is the main reason for the toxicity of Fuzi^26^. Here we showed that Fuzi can act on multiple targets that directly modulate the flow of Na^+^, Ca^2+^, and K^+^. The results demonstrated the reliability of our study.

### The compound and target differences for the efficacy and toxicity of Fuzi

**Figure 9:**
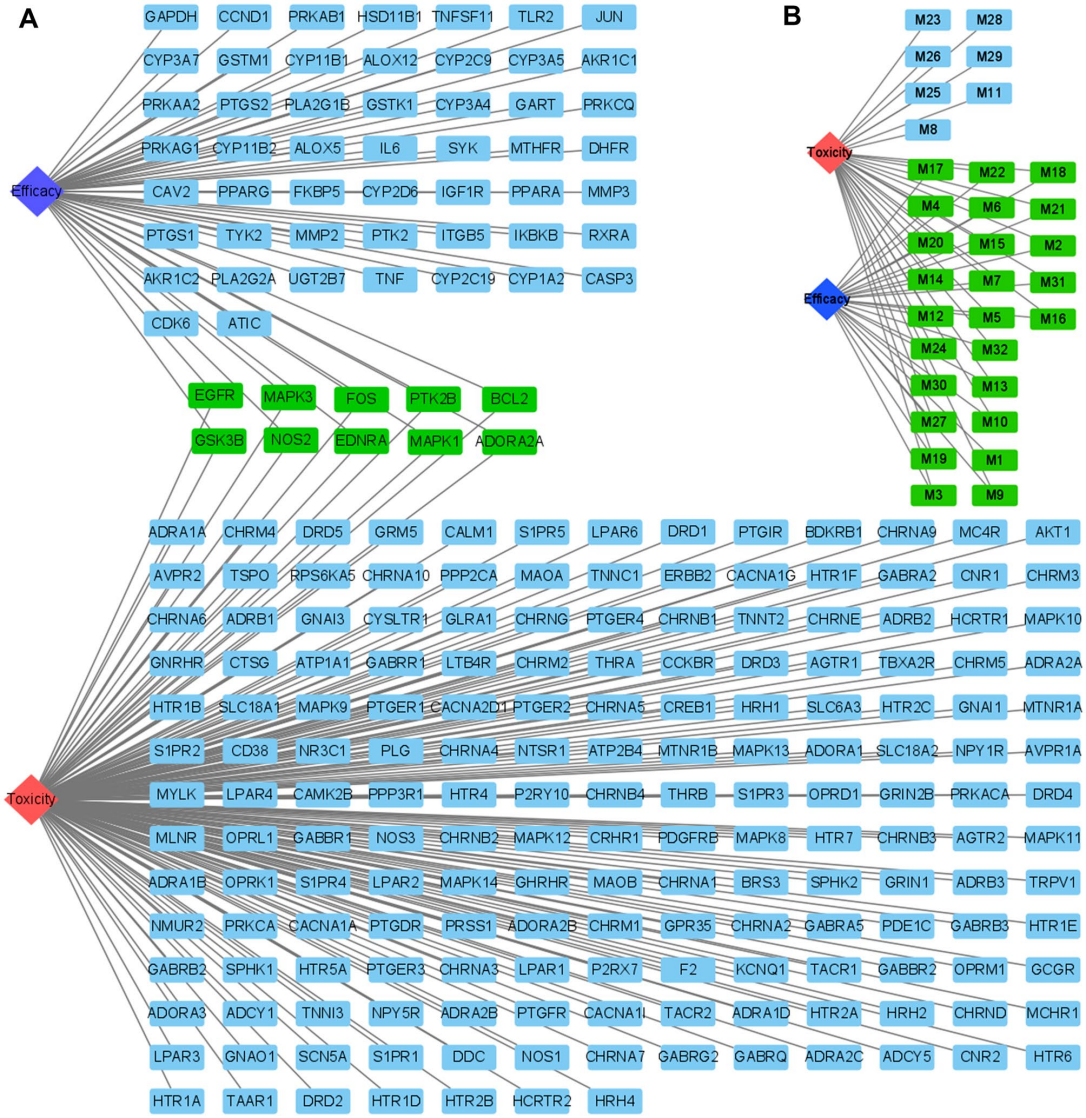
Comparison of the compounds and targets that are responsible for the toxicity and efficacy of Fuzi. The full name of compounds and targets are shown in Table 1 and Supplementary Table S1, respectively.

Since Fuzi is the typical Chinese herbal medicine with salient efficacy and strong toxicity, we then explored the mechanism difference between efficacy and toxicity. First, we compared the targets involved in inducing toxicity and targets inducing therapeutic effects on rheumatoid arthritis (Fig. 9A). The results showed that 51 targets only contribute to the efficacy in treating rheumatoid arthritis, 189 targets are only responsible for the toxicity, and 10 targets are involved in both toxicity and efficacy of Fuzi. Then, we explored the compounds involved in inducing toxicity and compounds contributing to the therapeutic effects on rheumatoid arthritis (Fig. 9B). The results showed that 7 compounds only contribute to the toxicity, 15 targets are involved in both toxicity and efficacy of Fuzi, and no compound is only associated with efficacy. The results indicated that the compounds and targets contributing to the efficacy are also responsible for the toxicity of Fuzi.

## Discussion

As the typical herbal medicine with strong toxicity and obvious efficacy against rheumatoid arthritis, Fuzi present in 13.20% of 500 well-known prescriptions used in clinical practice^30^. Although Fuzi has been in the spotlight for a long time from the researchers, the mechanism relationship between the toxicity and efficacy has remain not fully known for a long time. Using the efficacy in treating rheumatoid arthritis as an example, we adopted a systems pharmacology approach to screen the bioactive compounds and to identify the potential targets. We found out that one compound can act on multiple targets and different targets can link to the same compound, typifying the properties of herbal medicines that they can act in a holistic way to treat diseases. We also found out that 25 bioactive compounds can act on 61 targets and 27 pathways to treat rheumatoid arthritis, and 32 bioactive compounds can act on 187 targets and 4 pathways to induce toxicity.

Rheumatoid arthritis is a chronic and inflammatory autoimmune disease that causes symmetrical polyarthritis of large and small joints. In this disease, cytokines function in a network of overlapping, synergistic, antagonistic, and inhibitory ways to mediate the development and progress of disease^31^. Because of the importance of cytokines, effects have been made to develop cytokine-targeted therapies, such as therapies that target important proinflammatory cytokines TNF-α, IL-1, IL-6, IL-17, IL-20, IL-21, IL-23^32^. In our study, we found out that Fuzi can target proinflammatory cytokines associated genes including TNF and IL6 to ameliorate rheumatoid arthritis. In addition, Fuzi can target cytokines associated pathways such as TNF signaling pathway, NF-kappa B signaling pathway. The results indicate that modulation of inflammation state is one of the main mechanisms of Fuzi to treat rheumatoid arthritis.

The toxicity of Fuzi poses a great threat to the patients and can even lead to the death of patients. Studies have demonstrated that the toxicity of Fuzi mainly derives from diester diterpene alkaloids including aconitine, mesaconitine, and hypaconitine^11^. In our study, we found out that the toxicity of Fuzi was not only associated with diester diterpene alkaloids, but also all other compounds as well. The result did not contradict the clinical and animal studies since other diterpene alkaloids such as benzoylmesaconine are toxic as well^33^. In our study, we also found out that non-toxic compounds can act on the targets of toxic compounds. Taking adrenergic signaling in the cardiomyocytes pathway as an example. Delphin_qt (M5), benzoylnapelline (M18), denudatine (M20), songorine (M30) can act on AKT1, and AKT1, ATP1A1, ATP2B4, BCL2, and other targets in adrenergic signaling in cardiomyocytes pathway to induce toxicity. In addition, the compounds that are usually believed to be non-toxic also involved in the toxicity of Fuzi. For example, myristic acid, a compound widely used in the food industry as a flavor ingredient, does not pose a health risk to humans^34^. In our study, myristic acid can act on hypaconitine targets TRPV1, THRA, MAPK1, etc. However, whether this action can lead to increase of toxicity, or decrease of toxicity, or no change of toxicity is known. Further animal studies are needed to confirm the three possible results. Although the effects of non-toxic compounds on final toxicity of Fuzi is known, the results can add new knowledge for our understanding of the toxicity of herbal medicines, i.e., non-toxic compounds can act on targets of toxic compounds, and therefore may influence the toxicity of Fuzi.

In the clinic, the major way to avoid the toxicity of Fuzi is processing (*Paozhi*). The main processing method includes boiling Fuzi in water for a long period of time. In this process, the main toxic diester-diterpenoid alkaloids including aconitine, mesaconitine, and hypaconitine can be first transformed into monoester-diterpenoid alkaloids and finally into unesterified compounds^35^. The unesterified diterpenoid alkaloids showed no toxicity in the clinic, but at the same time, their pharmacological activities are not influenced^30^. Although Fuzi must be processed and boiled for a long time before orally taken, there are many cases of using unprocessed Fuzi and Fuzi that are not boiled for enough time^36^. In addition, many Fuzi victims used Fuzi in medicinal liquors. As a result, there are many cases that toxic alkaloids lead to the Fuzi poisoning in clinic^36^. Therefore, in our study, we collected the compounds of processed and raw Fuzi, including diester-diterpenoid alkaloids and unesterified compounds in our study to investigate the efficacy-toxicity relationship of Fuzi. We found out that non-toxic compounds can act on targets of toxic compounds and therefore may influence the toxicity. Clinically, the processed Fuzi showed almost no toxicity. Therefore, our study also indicates that boiling Fuzi in water for a long period of time is a necessary step to avoid the toxicity of Fuzi, and the non-toxic compounds alone cannot induce toxicity.

In our study, some drawbacks should be noted. Although many targets of Fuzi have been validated by literatures, many targets identified in this study are needed to be further validated by in vivo studies. In addition, system pharmacology is intrinsically flawed in several aspects. First, the efficacy and toxicity of a drug are directly related to the dosage, and systems pharmacology currently cannot give the direct dosage- phenotype outcome. Secondly, an herbal medicine can contain hundreds or even thousands of different compounds, and it is now not possible to identify all the compounds that exist in Fuzi. Therefore, not all bioactive compounds and targets in our study can be screened currently. Thirdly, herbal compounds can be metabolized by gut microbiota and liver, and the metabolite can act different targets compared with their parent compounds. Even though we have predicted the aglycones of glycosides in our study, not all the metabolites can be predicted based on the structure of compounds. Therefore, not all the bioactive metabolites in the body can be predicted as well. Nevertheless, as an efficient and new tool to study the mechanisms of herbal medicines systemically, systems pharmacology enables us to understand the relationships between efficacy and toxicity.

In recent years, gut microbiota has emerged as a new frontier to understand the development and progress of diseases. Dysbiosis of gut microbiota such as *Prevotella histicola* is involved in the pathogenesis of rheumatoid arthritis^37–40^. Gut microbiota can synthesis and release a large number of metabolites with anti-inflammatory effects such as short-chain fatty acids to ameliorate diseases^41–43^. In our study, some compounds in Fuzi are not classified as bioactive compounds according to our screen standard, such as arachic acid. However, arachic acid is reported to be able to modulate the composition of gut microbiota^44,45^. Therefore, other compounds that are omitted in our study may act on gut microbiota to ameliorate rheumatoid arthritis. In addition to small molecular compounds, big molecular compounds that mainly include polysaccharides are also the bioactive compounds of herbal medicine. Polysaccharides can modulate the composition of gut microbiota and can be metabolized into short-chain fatty acids to modulate the immune system of hosts^46–48^. Currently, the in silicon approach can only screen small molecules, and polysaccharides are usually omitted. Therefore, like other compounds such as arachic acid, polysaccharides of Fuzi may also be effective on rheumatoid arthritis, and further studies are needed to confirm this hypothesis.

## Conclusion

Many herbal medicines with toxicity are extensively used in clinic to treat diseases, however, the mechanism relationships between the toxicity and efficacy of herbal medicines remain unknown. Fuzi is the typical toxic herbal medicine with remarkable clinical efficacy. Using Fuzi in treating rheumatoid arthritis as an example, we demonstrated that the efficacy of Fuzi can be attributed to 25 bioactive compounds that act holistically on 61 targets and 27 pathways, and the toxicity of Fuzi can be attributed to 32 compounds that act holistically on 187 targets and 4 pathways. In addition, non-toxic compound such as myristic acid can act on targets of toxic compounds and therefore may influence the toxicity. However, the effects of non-toxic compounds on the final toxicity of Fuzi remain to be studied further. The results suggested that removing of toxic compounds by processing and boiling is a necessary procedure to avoid the toxicity of Fuzi.

## Supplementary Information

Supplementary Information.

## Data Availability

All relevant data are within the paper and its Supporting Information files.
