# Supplementary material for: Revealing the efficacy-toxicity relationship of Fuzi in treating rheumatoid arthritis by systems pharmacology
Source: Sci Rep. 2021 Nov 29;11:23083. doi: 10.1038/s41598-021-02167-5 (PMC8630009; doi:10.1038/s41598-021-02167-5)
Supplement: Supplementary file 1 — Supplementary Information. [file 41598_2021_2167_MOESM1_ESM.docx]

**Supplementary materials**

**Revealing the efficacy-toxicity relationship of Fuzi in treating rheumatoid arthritis by systems pharmacology**

Wuwen Feng^a,b,#^, Juan Liu^a,b,#^, Dandan Zhang^a^, Yuzhu Tan^a^, Hao Cheng^a,b^, Cheng Peng^a,b,⁎^

^a^ *State Key Laboratory of Southwestern Chinese Medicine Resources, School of Pharmacy, Chengdu University of Traditional Chinese Medicine, Chengdu 611130, China*

^b^ *Key Laboratory of the Ministry of Education for Standardization of Chinese Medicine, Chengdu University of Traditional Chinese Medicine, Chengdu 611130, China*

^#^ Wuwen Feng and Juan Liu contributed equally to this work.

^⁎^ Corresponding authors at: *School of Pharmacy, Chengdu University of Traditional Chinese Medicine, Chengdu 611130, China*.

E-mail addresses: pengchengcxy@126.com (Cheng Peng).


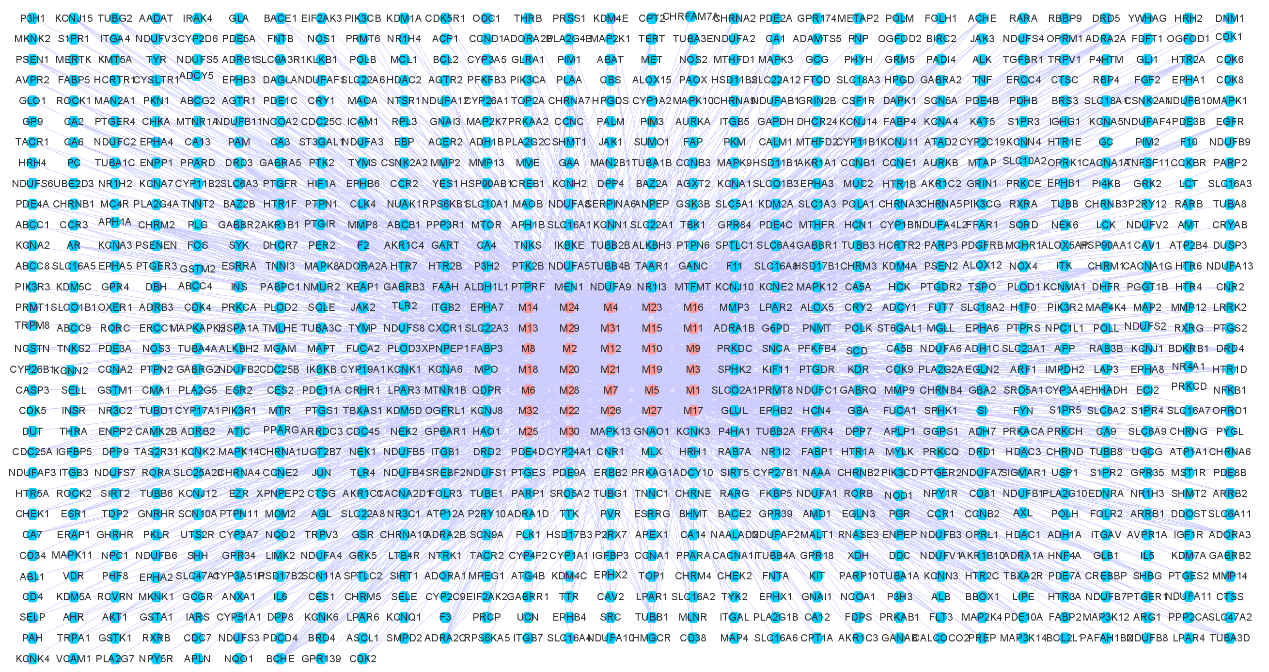


**Supplementary Fig. S1.** Compound-target network of all the bioactive compounds in Fuzi.

**Supplementary Table S1.** The detailed target genes of Fuzi.

| Gene symbol | Species | David Gene Name |
| --- | --- | --- |
| HTR4 | Homo sapiens | 5-hydroxytryptamine receptor 4 (HTR4) |
| MYLK | Homo sapiens | myosin light chain kinase (MYLK) |
| ALKBH3 | Homo sapiens | alkB homolog 3, alpha-ketoglutaratedependent dioxygenase (ALKBH3) |
| ALKBH2 | Homo sapiens | alkB homolog 2, alpha-ketoglutarate dependent dioxygenase (ALKBH2) |
| LIPE | Homo sapiens | lipase E, hormone sensitive type (LIPE) |
| HTR6 | Homo sapiens | 5-hydroxytryptamine receptor 6 (HTR6) |
| TBK1 | Homo sapiens | TANK binding kinase 1 (TBK1) |
| HTR7 | Homo sapiens | 5-hydroxytryptamine receptor 7 (HTR7) |
| CYP2D6 | Homo sapiens | cytochrome P450 family 2 subfamily D member 6 (CYP2D6) |
| AKT1 | Homo sapiens | AKT serine/threonine kinase 1 (AKT1) |
| PRKACA | Homo sapiens | protein kinase cAMP-activated catalytic subunit alpha (PRKACA) |
| GSTK1 | Homo sapiens | glutathione S-transferase kappa 1 (GSTK1) |
| CSNK2A1 | Homo sapiens | casein kinase 2 alpha 1 (CSNK2A1) |
| CACNA2D1 | Homo sapiens | calcium voltage-gated channel auxiliary subunit alpha2delta 1 (CACNA2D1) |
| CSNK2A2 | Homo sapiens | casein kinase 2 alpha 2 (CSNK2A2) |
| RNASE3 | Homo sapiens | ribonuclease A family member 3 (RNASE3) |
| PRKAB1 | Homo sapiens | protein kinase AMP-activated non-catalytic subunit beta 1 (PRKAB1) |
| AR | Homo sapiens | androgen receptor (AR) |
| RBP4 | Homo sapiens | retinol binding protein 4 (RBP4) |
| MTHFD1 | Homo sapiens | methylenetetrahydrofolate dehydrogenase, cyclohydrolase and formyltetrahydrofolate synthetase 1 (MTHFD1) |
| MTHFD2 | Homo sapiens | methylenetetrahydrofolate dehydrogenase (NADP+ dependent) 2, methenyltetrahydrofolate cyclohydrolase (MTHFD2) |
| AGTR1 | Homo sapiens | angiotensin II receptor type 1 (AGTR1) |
| AGTR2 | Homo sapiens | angiotensin II receptor type 2 (AGTR2) |
| PADI4 | Homo sapiens | peptidyl arginine deiminase 4 (PADI4) |
| ABCB1 | Homo sapiens | ATP binding cassette subfamily B member 1 (ABCB1) |
| SHMT2 | Homo sapiens | serine hydroxymethyltransferase 2 (SHMT2) |
| SHMT1 | Homo sapiens | serine hydroxymethyltransferase 1 (SHMT1) |
| BAZ2A | Homo sapiens | bromodomain adjacent to zinc finger domain 2A (BAZ2A) |
| SLC5A1 | Homo sapiens | solute carrier family 5 member 1 (SLC5A1) |
| PLA2G5 | Homo sapiens | phospholipase A2 group V (PLA2G5) |
| BAZ2B | Homo sapiens | bromodomain adjacent to zinc finger domain 2B (BAZ2B) |
| PLA2G7 | Homo sapiens | phospholipase A2 group VII (PLA2G7) |
| HSD11B1 | Homo sapiens | hydroxysteroid 11-beta dehydrogenase 1 (HSD11B1) |
| GHRHR | Homo sapiens | growth hormone releasing hormone receptor (GHRHR) |
| HSD11B2 | Homo sapiens | hydroxysteroid 11-beta dehydrogenase 2 (HSD11B2) |
| HAO1 | Homo sapiens | hydroxyacid oxidase 1 (HAO1) |
| GSTM2 | Homo sapiens | glutathione S-transferase mu 2 (GSTM2) |
| CREBBP | Homo sapiens | CREB binding protein (CREBBP) |
| JUN | Homo sapiens | Jun proto-oncogene, AP-1 transcription factor subunit (JUN) |
| GSTM1 | Homo sapiens | glutathione S-transferase mu 1 (GSTM1) |
| CYP4F2 | Homo sapiens | cytochrome P450 family 4 subfamily F member 2 (CYP4F2) |
| FUCA1 | Homo sapiens | fucosidase, alpha-L- 1, tissue (FUCA1) |
| FUCA2 | Homo sapiens | fucosidase, alpha-L- 2, plasma (FUCA2) |
| EIF2AK3 | Homo sapiens | eukaryotic translation initiation factor 2 alpha kinase 3 (EIF2AK3) |
| EIF2AK2 | Homo sapiens | eukaryotic translation initiation factor 2 alpha kinase 2 (EIF2AK2) |
| GRIN2B | Homo sapiens | glutamate ionotropic receptor NMDA type subunit 2B (GRIN2B) |
| DHFR | Homo sapiens | dihydrofolate reductase (DHFR) |
| CYP2C9 | Homo sapiens | cytochrome P450 family 2 subfamily C member 9 (CYP2C9) |
| BHMT | Homo sapiens | betaine--homocysteine S-methyltransferase (BHMT) |
| TDP2 | Homo sapiens | tyrosyl-DNA phosphodiesterase 2 (TDP2) |
| NOX4 | Homo sapiens | NADPH oxidase 4 (NOX4) |
| GPR18 | Homo sapiens | G protein-coupled receptor 18 (GPR18) |
| ACHE | Homo sapiens | acetylcholinesterase (Cartwright blood group) (ACHE) |
| ARF1 | Homo sapiens | ADP ribosylation factor 1 (ARF1) |
| PRKAG1 | Homo sapiens | protein kinase AMP-activated non-catalytic subunit gamma 1 (PRKAG1) |
| IKBKB | Homo sapiens | inhibitor of kappa light polypeptide gene enhancer in B-cells, kinase beta (IKBKB) |
| SMPD2 | Homo sapiens | sphingomyelin phosphodiesterase 2 (SMPD2) |
| TUBA1C | Homo sapiens | tubulin alpha 1c (TUBA1C) |
| TUBA1B | Homo sapiens | tubulin alpha 1b (TUBA1B) |
| SHH | Homo sapiens | sonic hedgehog (SHH) |
| ATIC | Homo sapiens | 5-aminoimidazole-4-carboxamide ribonucleotide formyltransferase/IMP cyclohydrolase (ATIC) |
| TUBA1A | Homo sapiens | tubulin alpha 1a (TUBA1A) |
| TSPO | Homo sapiens | translocator protein (TSPO) |
| JAK2 | Homo sapiens | Janus kinase 2 (JAK2) |
| SLC18A1 | Homo sapiens | solute carrier family 18 member A1 (SLC18A1) |
| IKBKE | Homo sapiens | inhibitor of kappa light polypeptide gene enhancer in B-cells, kinase epsilon (IKBKE) |
| JAK3 | Homo sapiens | Janus kinase 3 (JAK3) |
| SLC18A2 | Homo sapiens | solute carrier family 18 member A2 (SLC18A2) |
| SLC18A3 | Homo sapiens | solute carrier family 18 member A3 (SLC18A3) |
| JAK1 | Homo sapiens | Janus kinase 1 (JAK1) |
| GPR39 | Homo sapiens | G protein-coupled receptor 39 (GPR39) |
| DUSP3 | Homo sapiens | dual specificity phosphatase 3 (DUSP3) |
| GPR34 | Homo sapiens | G protein-coupled receptor 34 (GPR34) |
| ARG1 | Homo sapiens | arginase 1 (ARG1) |
| GPR35 | Homo sapiens | G protein-coupled receptor 35 (GPR35) |
| SPHK2 | Homo sapiens | sphingosine kinase 2 (SPHK2) |
| SPHK1 | Homo sapiens | sphingosine kinase 1 (SPHK1) |
| SIRT5 | Homo sapiens | sirtuin 5 (SIRT5) |
| FOS | Homo sapiens | Fos proto-oncogene, AP-1 transcription factor subunit (FOS) |
| F2 | Homo sapiens | coagulation factor II, thrombin (F2) |
| F3 | Homo sapiens | coagulation factor III, tissue factor (F3) |
| SIRT1 | Homo sapiens | sirtuin 1 (SIRT1) |
| SIRT2 | Homo sapiens | sirtuin 2 (SIRT2) |
| MMP12 | Homo sapiens | matrix metallopeptidase 12 (MMP12) |
| MMP14 | Homo sapiens | matrix metallopeptidase 14 (MMP14) |
| MMP13 | Homo sapiens | matrix metallopeptidase 13 (MMP13) |
| KCNMA1 | Homo sapiens | potassium calcium-activated channel subfamily M alpha 1 (KCNMA1) |
| PPARG | Homo sapiens | peroxisome proliferator activated receptor gamma (PPARG) |
| PPARA | Homo sapiens | peroxisome proliferator activated receptor alpha (PPARA) |
| CRYAB | Homo sapiens | crystallin alpha B (CRYAB) |
| PAFAH1B2 | Homo sapiens | platelet activating factor acetylhydrolase 1b catalytic subunit 2 (PAFAH1B2) |
| PPARD | Homo sapiens | peroxisome proliferator activated receptor delta (PPARD) |
| KCNA1 | Homo sapiens | potassium voltage-gated channel subfamily A member 1 (KCNA1) |
| ODC1 | Homo sapiens | ornithine decarboxylase 1 (ODC1) |
| KCNA2 | Homo sapiens | potassium voltage-gated channel subfamily A member 2 (KCNA2) |
| KCNA3 | Homo sapiens | potassium voltage-gated channel subfamily A member 3 (KCNA3) |
| KCNA4 | Homo sapiens | potassium voltage-gated channel subfamily A member 4 (KCNA4) |
| KCNA5 | Homo sapiens | potassium voltage-gated channel subfamily A member 5 (KCNA5) |
| KCNA6 | Homo sapiens | potassium voltage-gated channel subfamily A member 6 (KCNA6) |
| PTGS2 | Homo sapiens | prostaglandin-endoperoxide synthase 2 (PTGS2) |
| KCNA7 | Homo sapiens | potassium voltage-gated channel subfamily A member 7 (KCNA7) |
| EGFR | Homo sapiens | epidermal growth factor receptor (EGFR) |
| PTGS1 | Homo sapiens | prostaglandin-endoperoxide synthase 1 (PTGS1) |
| TUBA3E | Homo sapiens | tubulin alpha 3e (TUBA3E) |
| GLRA1 | Homo sapiens | glycine receptor alpha 1 (GLRA1) |
| TUBA3D | Homo sapiens | tubulin alpha 3d (TUBA3D) |
| TUBA3C | Homo sapiens | tubulin alpha 3c (TUBA3C) |
| GC | Homo sapiens | GC, vitamin D binding protein (GC) |
| P4HTM | Homo sapiens | prolyl 4-hydroxylase, transmembrane (P4HTM) |
| P2RY12 | Homo sapiens | purinergic receptor P2Y12 (P2RY12) |
| P2RY10 | Homo sapiens | purinergic receptor P2Y10 (P2RY10) |
| EGLN3 | Homo sapiens | egl-9 family hypoxia inducible factor 3 (EGLN3) |
| SLC16A1 | Homo sapiens | solute carrier family 16 member 1 (SLC16A1) |
| PKLR | Homo sapiens | pyruvate kinase, liver and RBC (PKLR) |
| AADAT | Homo sapiens | aminoadipate aminotransferase (AADAT) |
| ATAD2 | Homo sapiens | ATPase family, AAA domain containing 2 (ATAD2) |
| BBOX1 | Homo sapiens | gamma-butyrobetaine hydroxylase 1 (BBOX1) |
| FABP1 | Homo sapiens | fatty acid binding protein 1 (FABP1) |
| FABP2 | Homo sapiens | fatty acid binding protein 2 (FABP2) |
| FABP3 | Homo sapiens | fatty acid binding protein 3 (FABP3) |
| FABP4 | Homo sapiens | fatty acid binding protein 4 (FABP4) |
| FABP5 | Homo sapiens | fatty acid binding protein 5 (FABP5) |
| APEX1 | Homo sapiens | apurinic/apyrimidinic endodeoxyribonuclease 1 (APEX1) |
| PKN1 | Homo sapiens | protein kinase N1 (PKN1) |
| ALDH1L1 | Homo sapiens | aldehyde dehydrogenase 1 family member L1 (ALDH1L1) |
| UBE2D3 | Homo sapiens | ubiquitin conjugating enzyme E2 D3 (UBE2D3) |
| RORC | Homo sapiens | RAR related orphan receptor C (RORC) |
| PLOD3 | Homo sapiens | procollagen-lysine,2-oxoglutarate 5-dioxygenase 3 (PLOD3) |
| PLOD2 | Homo sapiens | procollagen-lysine,2-oxoglutarate 5-dioxygenase 2 (PLOD2) |
| RORA | Homo sapiens | RAR related orphan receptor A (RORA) |
| PLOD1 | Homo sapiens | procollagen-lysine,2-oxoglutarate 5-dioxygenase 1 (PLOD1) |
| RORB | Homo sapiens | RAR related orphan receptor B (RORB) |
| ATP12A | Homo sapiens | ATPase H+/K+ transporting non-gastric alpha2 subunit (ATP12A) |
| HPGDS | Homo sapiens | hematopoietic prostaglandin D synthase (HPGDS) |
| FNTA | Homo sapiens | farnesyltransferase, CAAX box, alpha (FNTA) |
| ADORA3 | Homo sapiens | adenosine A3 receptor (ADORA3) |
| FNTB | Homo sapiens | farnesyltransferase, CAAX box, beta (FNTB) |
| ADORA1 | Homo sapiens | adenosine A1 receptor (ADORA1) |
| SLC16A6 | Homo sapiens | solute carrier family 16 member 6 (SLC16A6) |
| PIM1 | Homo sapiens | Pim-1 proto-oncogene, serine/threonine kinase (PIM1) |
| NEK1 | Homo sapiens | NIMA related kinase 1 (NEK1) |
| SLC16A7 | Homo sapiens | solute carrier family 16 member 7 (SLC16A7) |
| NEK2 | Homo sapiens | NIMA related kinase 2 (NEK2) |
| SLC16A8 | Homo sapiens | solute carrier family 16 member 8 (SLC16A8) |
| PIM3 | Homo sapiens | Pim-3 proto-oncogene, serine/threonine kinase (PIM3) |
| PIM2 | Homo sapiens | Pim-2 proto-oncogene, serine/threonine kinase (PIM2) |
| KMT5A | Homo sapiens | lysine methyltransferase 5A (KMT5A) |
| SLC16A2 | Homo sapiens | solute carrier family 16 member 2 (SLC16A2) |
| SLC16A3 | Homo sapiens | solute carrier family 16 member 3 (SLC16A3) |
| SLC16A4 | Homo sapiens | solute carrier family 16 member 4 (SLC16A4) |
| SLC16A5 | Homo sapiens | solute carrier family 16 member 5 (SLC16A5) |
| KCNH2 | Homo sapiens | potassium voltage-gated channel subfamily H member 2 (KCNH2) |
| CDC25C | Homo sapiens | cell division cycle 25C (CDC25C) |
| CDC25A | Homo sapiens | cell division cycle 25A (CDC25A) |
| TUBA4A | Homo sapiens | tubulin alpha 4a (TUBA4A) |
| CDC25B | Homo sapiens | cell division cycle 25B (CDC25B) |
| NAAA | Homo sapiens | N-acylethanolamine acid amidase (NAAA) |
| MAPKAPK2 | Homo sapiens | mitogen-activated protein kinase-activated protein kinase 2 (MAPKAPK2) |
| PFKFB4 | Homo sapiens | 6-phosphofructo-2-kinase/fructose-2,6-biphosphatase 4 (PFKFB4) |
| PFKFB3 | Homo sapiens | 6-phosphofructo-2-kinase/fructose-2,6-biphosphatase 3 (PFKFB3) |
| KCNE2 | Homo sapiens | potassium voltage-gated channel subfamily E regulatory subunit 2 (KCNE2) |
| GPR84 | Homo sapiens | G protein-coupled receptor 84 (GPR84) |
| CYP2C19 | Homo sapiens | cytochrome P450 family 2 subfamily C member 19 (CYP2C19) |
| CYP19A1 | Homo sapiens | cytochrome P450 family 19 subfamily A member 1 (CYP19A1) |
| SLCO1B1 | Homo sapiens | solute carrier organic anion transporter family member 1B1 (SLCO1B1) |
| SLC9A3R1 | Homo sapiens | SLC9A3 regulator 1 (SLC9A3R1) |
| SRD5A2 | Homo sapiens | steroid 5 alpha-reductase 2 (SRD5A2) |
| HTR1E | Homo sapiens | 5-hydroxytryptamine receptor 1E (HTR1E) |
| SRD5A1 | Homo sapiens | steroid 5 alpha-reductase 1 (SRD5A1) |
| HTR1F | Homo sapiens | 5-hydroxytryptamine receptor 1F (HTR1F) |
| AGXT2 | Homo sapiens | alanine--glyoxylate aminotransferase 2 (AGXT2) |
| NEK6 | Homo sapiens | NIMA related kinase 6 (NEK6) |
| HTR1D | Homo sapiens | 5-hydroxytryptamine receptor 1D (HTR1D) |
| HTR1A | Homo sapiens | 5-hydroxytryptamine receptor 1A (HTR1A) |
| HTR1B | Homo sapiens | 5-hydroxytryptamine receptor 1B (HTR1B) |
| NFKB1 | Homo sapiens | nuclear factor kappa B subunit 1 (NFKB1) |
| TBXAS1 | Homo sapiens | thromboxane A synthase 1 (TBXAS1) |
| MDM2 | Homo sapiens | MDM2 proto-oncogene (MDM2) |
| SHBG | Homo sapiens | sex hormone binding globulin (SHBG) |
| ITGB1 | Homo sapiens | integrin subunit beta 1 (ITGB1) |
| KCNK6 | Homo sapiens | potassium two pore domain channel subfamily K member 6 (KCNK6) |
| HSP90AB1 | Homo sapiens | heat shock protein 90 alpha family class B member 1 (HSP90AB1) |
| ITGB5 | Homo sapiens | integrin subunit beta 5 (ITGB5) |
| FLT3 | Homo sapiens | fms related tyrosine kinase 3 (FLT3) |
| LRRK2 | Homo sapiens | leucine rich repeat kinase 2 (LRRK2) |
| ITGB3 | Homo sapiens | integrin subunit beta 3 (ITGB3) |
| ITGB2 | Homo sapiens | integrin subunit beta 2 (ITGB2) |
| HTR2B | Homo sapiens | 5-hydroxytryptamine receptor 2B (HTR2B) |
| PIK3CD | Homo sapiens | phosphatidylinositol-4,5-bisphosphate 3-kinase catalytic subunit delta (PIK3CD) |
| HTR2C | Homo sapiens | 5-hydroxytryptamine receptor 2C (HTR2C) |
| ARRB1 | Homo sapiens | arrestin beta 1 (ARRB1) |
| ARRB2 | Homo sapiens | arrestin beta 2 (ARRB2) |
| PIK3CB | Homo sapiens | phosphatidylinositol-4,5-bisphosphate 3-kinase catalytic subunit beta (PIK3CB) |
| HTR2A | Homo sapiens | 5-hydroxytryptamine receptor 2A (HTR2A) |
| ITGAL | Homo sapiens | integrin subunit alpha L (ITGAL) |
| PIK3CG | Homo sapiens | phosphatidylinositol-4,5-bisphosphate 3-kinase catalytic subunit gamma (PIK3CG) |
| SUMO1 | Homo sapiens | small ubiquitin-like modifier 1 (SUMO1) |
| ITGAV | Homo sapiens | integrin subunit alpha V (ITGAV) |
| ITGB7 | Homo sapiens | integrin subunit beta 7 (ITGB7) |
| ACP1 | Homo sapiens | acid phosphatase 1, soluble (ACP1) |
| HSP90AA1 | Homo sapiens | heat shock protein 90 alpha family class A member 1 (HSP90AA1) |
| ITGA4 | Homo sapiens | integrin subunit alpha 4 (ITGA4) |
| SIGMAR1 | Homo sapiens | sigma non-opioid intracellular receptor 1 (SIGMAR1) |
| NDUFC2 | Homo sapiens | NADH:ubiquinone oxidoreductase subunit C2 (NDUFC2) |
| NDUFC1 | Homo sapiens | NADH:ubiquinone oxidoreductase subunit C1 (NDUFC1) |
| HTR3A | Homo sapiens | 5-hydroxytryptamine receptor 3A (HTR3A) |
| DDOST | Homo sapiens | dolichyl-diphosphooligosaccharide--protein glycosyltransferase non-catalytic subunit (DDOST) |
| ADORA2A | Homo sapiens | adenosine A2a receptor (ADORA2A) |
| NPC1 | Homo sapiens | NPC intracellular cholesterol transporter 1 (NPC1) |
| PIK3CA | Homo sapiens | phosphatidylinositol-4,5-bisphosphate 3-kinase catalytic subunit alpha (PIK3CA) |
| ADORA2B | Homo sapiens | adenosine A2b receptor (ADORA2B) |
| TOP1 | Homo sapiens | topoisomerase (DNA) I (TOP1) |
| MET | Homo sapiens | MET proto-oncogene, receptor tyrosine kinase (MET) |
| GAPDH | Homo sapiens | glyceraldehyde-3-phosphate dehydrogenase (GAPDH) |
| BIRC2 | Homo sapiens | baculoviral IAP repeat containing 2 (BIRC2) |
| UGT2B7 | Homo sapiens | UDP glucuronosyltransferase family 2 member B7 (UGT2B7) |
| SLC47A2 | Homo sapiens | solute carrier family 47 member 2 (SLC47A2) |
| NDUFB9 | Homo sapiens | NADH:ubiquinone oxidoreductase subunit B9 (NDUFB9) |
| NDUFB8 | Homo sapiens | NADH:ubiquinone oxidoreductase subunit B8 (NDUFB8) |
| NDUFB7 | Homo sapiens | NADH:ubiquinone oxidoreductase subunit B7 (NDUFB7) |
| PRKAA2 | Homo sapiens | protein kinase AMP-activated catalytic subunit alpha 2 (PRKAA2) |
| SLC47A1 | Homo sapiens | solute carrier family 47 member 1 (SLC47A1) |
| NDUFB6 | Homo sapiens | NADH:ubiquinone oxidoreductase subunit B6 (NDUFB6) |
| ROCK1 | Homo sapiens | Rho associated coiled-coil containing protein kinase 1 (ROCK1) |
| SRC | Homo sapiens | SRC proto-oncogene, non-receptor tyrosine kinase (SRC) |
| NDUFB5 | Homo sapiens | NADH:ubiquinone oxidoreductase subunit B5 (NDUFB5) |
| ROCK2 | Homo sapiens | Rho associated coiled-coil containing protein kinase 2 (ROCK2) |
| NDUFB4 | Homo sapiens | NADH:ubiquinone oxidoreductase subunit B4 (NDUFB4) |
| NDUFB3 | Homo sapiens | NADH:ubiquinone oxidoreductase subunit B3 (NDUFB3) |
| NDUFB2 | Homo sapiens | NADH:ubiquinone oxidoreductase subunit B2 (NDUFB2) |
| NDUFB1 | Homo sapiens | NADH:ubiquinone oxidoreductase subunit B1 (NDUFB1) |
| PRCP | Homo sapiens | prolylcarboxypeptidase (PRCP) |
| MALT1 | Homo sapiens | MALT1 paracaspase (MALT1) |
| GANAB | Homo sapiens | glucosidase II alpha subunit (GANAB) |
| CDC45 | Homo sapiens | cell division cycle 45 (CDC45) |
| FYN | Homo sapiens | FYN proto-oncogene, Src family tyrosine kinase (FYN) |
| SLC25A20 | Homo sapiens | solute carrier family 25 member 20 (SLC25A20) |
| KCNJ1 | Homo sapiens | potassium voltage-gated channel subfamily J member 1 (KCNJ1) |
| NDUFA9 | Homo sapiens | NADH:ubiquinone oxidoreductase subunit A9 (NDUFA9) |
| NTRK1 | Homo sapiens | neurotrophic receptor tyrosine kinase 1 (NTRK1) |
| NDUFA8 | Homo sapiens | NADH:ubiquinone oxidoreductase subunit A8 (NDUFA8) |
| PTPN1 | Homo sapiens | protein tyrosine phosphatase, non-receptor type 1 (PTPN1) |
| NDUFA7 | Homo sapiens | NADH:ubiquinone oxidoreductase subunit A7 (NDUFA7) |
| DUT | Homo sapiens | deoxyuridine triphosphatase (DUT) |
| NDUFA6 | Homo sapiens | NADH:ubiquinone oxidoreductase subunit A6 (NDUFA6) |
| NDUFA5 | Homo sapiens | NADH:ubiquinone oxidoreductase subunit A5 (NDUFA5) |
| KCNJ8 | Homo sapiens | potassium voltage-gated channel subfamily J member 8 (KCNJ8) |
| NDUFA4 | Homo sapiens | NDUFA4, mitochondrial complex associated (NDUFA4) |
| NDUFA3 | Homo sapiens | NADH:ubiquinone oxidoreductase subunit A3 (NDUFA3) |
| ERAP1 | Homo sapiens | endoplasmic reticulum aminopeptidase 1 (ERAP1) |
| NDUFA2 | Homo sapiens | NADH:ubiquinone oxidoreductase subunit A2 (NDUFA2) |
| NDUFA1 | Homo sapiens | NADH:ubiquinone oxidoreductase subunit A1 (NDUFA1) |
| MERTK | Homo sapiens | MER proto-oncogene, tyrosine kinase (MERTK) |
| HTR5A | Homo sapiens | 5-hydroxytryptamine receptor 5A (HTR5A) |
| HCRTR2 | Homo sapiens | hypocretin receptor 2 (HCRTR2) |
| APLN | Homo sapiens | apelin (APLN) |
| HCRTR1 | Homo sapiens | hypocretin receptor 1 (HCRTR1) |
| PC | Homo sapiens | pyruvate carboxylase (PC) |
| TMLHE | Homo sapiens | trimethyllysine hydroxylase, epsilon (TMLHE) |
| PDCD4 | Homo sapiens | programmed cell death 4 (neoplastic transformation inhibitor) (PDCD4) |
| PTPN6 | Homo sapiens | protein tyrosine phosphatase, non-receptor type 6 (PTPN6) |
| KCNK1 | Homo sapiens | potassium two pore domain channel subfamily K member 1 (KCNK1) |
| KCNK2 | Homo sapiens | potassium two pore domain channel subfamily K member 2 (KCNK2) |
| PTPN2 | Homo sapiens | protein tyrosine phosphatase, non-receptor type 2 (PTPN2) |
| KCNK3 | Homo sapiens | potassium two pore domain channel subfamily K member 3 (KCNK3) |
| KCNK4 | Homo sapiens | potassium two pore domain channel subfamily K member 4 (KCNK4) |
| EPHB6 | Homo sapiens | EPH receptor B6 (EPHB6) |
| SLC23A1 | Homo sapiens | solute carrier family 23 member 1 (SLC23A1) |
| ECI2 | Homo sapiens | enoyl-CoA delta isomerase 2 (ECI2) |
| PREP | Homo sapiens | prolyl endopeptidase (PREP) |
| KEAP1 | Homo sapiens | kelch like ECH associated protein 1 (KEAP1) |
| CCNC | Homo sapiens | cyclin C (CCNC) |
| ICAM1 | Homo sapiens | intercellular adhesion molecule 1 (ICAM1) |
| ANPEP | Homo sapiens | alanyl aminopeptidase, membrane (ANPEP) |
| OGFOD1 | Homo sapiens | 2-oxoglutarate and iron dependent oxygenase domain containing 1 (OGFOD1) |
| OGFOD2 | Homo sapiens | 2-oxoglutarate and iron dependent oxygenase domain containing 2 (OGFOD2) |
| KDR | Homo sapiens | kinase insert domain receptor (KDR) |
| TNFSF11 | Homo sapiens | tumor necrosis factor superfamily member 11 (TNFSF11) |
| OXER1 | Homo sapiens | oxoeicosanoid receptor 1 (OXER1) |
| EPHB2 | Homo sapiens | EPH receptor B2 (EPHB2) |
| EPHB1 | Homo sapiens | EPH receptor B1 (EPHB1) |
| EPHB4 | Homo sapiens | EPH receptor B4 (EPHB4) |
| EPHB3 | Homo sapiens | EPH receptor B3 (EPHB3) |
| EPHA5 | Homo sapiens | EPH receptor A5 (EPHA5) |
| EPHA4 | Homo sapiens | EPH receptor A4 (EPHA4) |
| ST6GAL1 | Homo sapiens | ST6 beta-galactoside alpha-2,6-sialyltransferase 1 (ST6GAL1) |
| EPHA7 | Homo sapiens | EPH receptor A7 (EPHA7) |
| EPHA6 | Homo sapiens | EPH receptor A6 (EPHA6) |
| EPHA8 | Homo sapiens | EPH receptor A8 (EPHA8) |
| KCNQ1 | Homo sapiens | potassium voltage-gated channel subfamily Q member 1 (KCNQ1) |
| LAP3 | Homo sapiens | leucine aminopeptidase 3 (LAP3) |
| EPHA1 | Homo sapiens | EPH receptor A1 (EPHA1) |
| CES2 | Homo sapiens | carboxylesterase 2 (CES2) |
| EPHA3 | Homo sapiens | EPH receptor A3 (EPHA3) |
| CES1 | Homo sapiens | carboxylesterase 1 (CES1) |
| EPHA2 | Homo sapiens | EPH receptor A2 (EPHA2) |
| LPAR1 | Homo sapiens | lysophosphatidic acid receptor 1 (LPAR1) |
| PIK3R3 | Homo sapiens | phosphoinositide-3-kinase regulatory subunit 3 (PIK3R3) |
| LPAR2 | Homo sapiens | lysophosphatidic acid receptor 2 (LPAR2) |
| LPAR3 | Homo sapiens | lysophosphatidic acid receptor 3 (LPAR3) |
| PIK3R2 | Homo sapiens | phosphoinositide-3-kinase regulatory subunit 2 (PIK3R2) |
| MST1R | Homo sapiens | macrophage stimulating 1 receptor (MST1R) |
| LPAR4 | Homo sapiens | lysophosphatidic acid receptor 4 (LPAR4) |
| PIK3R1 | Homo sapiens | phosphoinositide-3-kinase regulatory subunit 1 (PIK3R1) |
| APH1A | Homo sapiens | aph-1 homolog A, gamma-secretase subunit (APH1A) |
| APH1B | Homo sapiens | aph-1 homolog B, gamma-secretase subunit (APH1B) |
| SI | Homo sapiens | sucrase-isomaltase (SI) |
| KCNN1 | Homo sapiens | potassium calcium-activated channel subfamily N member 1 (KCNN1) |
| KCNN2 | Homo sapiens | potassium calcium-activated channel subfamily N member 2 (KCNN2) |
| KCNN3 | Homo sapiens | potassium calcium-activated channel subfamily N member 3 (KCNN3) |
| KCNN4 | Homo sapiens | potassium calcium-activated channel subfamily N member 4 (KCNN4) |
| CCR1 | Homo sapiens | C-C motif chemokine receptor 1 (CCR1) |
| YES1 | Homo sapiens | YES proto-oncogene 1, Src family tyrosine kinase (YES1) |
| TAS2R31 | Homo sapiens | taste 2 receptor member 31 (TAS2R31) |
| SLC10A1 | Homo sapiens | solute carrier family 10 member 1 (SLC10A1) |
| SLC10A2 | Homo sapiens | solute carrier family 10 member 2 (SLC10A2) |
| PTK2 | Homo sapiens | protein tyrosine kinase 2 (PTK2) |
| ACER2 | Homo sapiens | alkaline ceramidase 2 (ACER2) |
| POLA1 | Homo sapiens | DNA polymerase alpha 1, catalytic subunit (POLA1) |
| CYP26A1 | Homo sapiens | cytochrome P450 family 26 subfamily A member 1 (CYP26A1) |
| LPAR6 | Homo sapiens | lysophosphatidic acid receptor 6 (LPAR6) |
| NDUFAF4 | Homo sapiens | NADH:ubiquinone oxidoreductase complex assembly factor 4 (NDUFAF4) |
| NDUFAF2 | Homo sapiens | NADH:ubiquinone oxidoreductase complex assembly factor 2 (NDUFAF2) |
| NDUFAF3 | Homo sapiens | NADH:ubiquinone oxidoreductase complex assembly factor 3 (NDUFAF3) |
| NDUFAF1 | Homo sapiens | NADH:ubiquinone oxidoreductase complex assembly factor 1 (NDUFAF1) |
| CALM1 | Homo sapiens | calmodulin 1 (CALM1) |
| PTPRS | Homo sapiens | protein tyrosine phosphatase, receptor type S (PTPRS) |
| PTGDR2 | Homo sapiens | prostaglandin D2 receptor 2 (PTGDR2) |
| AKR1B1 | Homo sapiens | aldo-keto reductase family 1 member B (AKR1B1) |
| KIF11 | Homo sapiens | kinesin family member 11 (KIF11) |
| CTSS | Homo sapiens | cathepsin S (CTSS) |
| PTPRF | Homo sapiens | protein tyrosine phosphatase, receptor type F (PTPRF) |
| MC4R | Homo sapiens | melanocortin 4 receptor (MC4R) |
| SPTLC1 | Homo sapiens | serine palmitoyltransferase long chain base subunit 1 (SPTLC1) |
| SPTLC2 | Homo sapiens | serine palmitoyltransferase long chain base subunit 2 (SPTLC2) |
| HNF4A | Homo sapiens | hepatocyte nuclear factor 4 alpha (HNF4A) |
| GNRHR | Homo sapiens | gonadotropin releasing hormone receptor (GNRHR) |
| MLX | Homo sapiens | MLX, MAX dimerization protein (MLX) |
| CD38 | Homo sapiens | CD38 molecule (CD38) |
| CTSG | Homo sapiens | cathepsin G (CTSG) |
| CD34 | Homo sapiens | CD34 molecule (CD34) |
| CTSC | Homo sapiens | cathepsin C (CTSC) |
| CCR3 | Homo sapiens | C-C motif chemokine receptor 3 (CCR3) |
| CCR2 | Homo sapiens | C-C motif chemokine receptor 2 (CCR2) |
| NCOA1 | Homo sapiens | nuclear receptor coactivator 1 (NCOA1) |
| NCOA2 | Homo sapiens | nuclear receptor coactivator 2 (NCOA2) |
| MME | Homo sapiens | membrane metalloendopeptidase (MME) |
| SYK | Homo sapiens | spleen associated tyrosine kinase (SYK) |
| GAA | Homo sapiens | glucosidase alpha, acid (GAA) |
| AKR1A1 | Homo sapiens | aldo-keto reductase family 1 member A1 (AKR1A1) |
| SREBF2 | Homo sapiens | sterol regulatory element binding transcription factor 2 (SREBF2) |
| KIT | Homo sapiens | KIT proto-oncogene receptor tyrosine kinase (KIT) |
| PABPC1 | Homo sapiens | poly (A) binding protein cytoplasmic 1 (PABPC1) |
| GBA | Homo sapiens | glucosylceramidase beta (GBA) |
| GNAI3 | Homo sapiens | G protein subunit alpha i3 (GNAI3) |
| HSD17B3 | Homo sapiens | hydroxysteroid 17-beta dehydrogenase 3 (HSD17B3) |
| GNAI1 | Homo sapiens | G protein subunit alpha i1 (GNAI1) |
| CYP17A1 | Homo sapiens | cytochrome P450 family 17 subfamily A member 1 (CYP17A1) |
| RXRB | Homo sapiens | retinoid X receptor beta (RXRB) |
| RXRA | Homo sapiens | retinoid X receptor alpha (RXRA) |
| HSD17B1 | Homo sapiens | hydroxysteroid 17-beta dehydrogenase 1 (HSD17B1) |
| HSD17B2 | Homo sapiens | hydroxysteroid 17-beta dehydrogenase 2 (HSD17B2) |
| PGGT1B | Homo sapiens | protein geranylgeranyltransferase type I subunit beta (PGGT1B) |
| CHRNA10 | Homo sapiens | cholinergic receptor nicotinic alpha 10 subunit (CHRNA10) |
| RXRG | Homo sapiens | retinoid X receptor gamma (RXRG) |
| XDH | Homo sapiens | xanthine dehydrogenase (XDH) |
| GABBR2 | Homo sapiens | gamma-aminobutyric acid type B receptor subunit 2 (GABBR2) |
| GABBR1 | Homo sapiens | gamma-aminobutyric acid type B receptor subunit 1 (GABBR1) |
| GCGR | Homo sapiens | glucagon receptor (GCGR) |
| AKR1C1 | Homo sapiens | aldo-keto reductase family 1 member C1 (AKR1C1) |
| AKR1C3 | Homo sapiens | aldo-keto reductase family 1 member C3 (AKR1C3) |
| AKR1C2 | Homo sapiens | aldo-keto reductase family 1 member C2 (AKR1C2) |
| GCG | Homo sapiens | glucagon (GCG) |
| AKR1C4 | Homo sapiens | aldo-keto reductase family 1 member C4 (AKR1C4) |
| GRIN1 | Homo sapiens | glutamate ionotropic receptor NMDA type subunit 1 (GRIN1) |
| PER2 | Homo sapiens | period circadian clock 2 (PER2) |
| NDUFAB1 | Homo sapiens | NADH:ubiquinone oxidoreductase subunit AB1 (NDUFAB1) |
| CD81 | Homo sapiens | CD81 molecule (CD81) |
| NR3C1 | Homo sapiens | nuclear receptor subfamily 3 group C member 1 (NR3C1) |
| MPO | Homo sapiens | myeloperoxidase (MPO) |
| NR3C2 | Homo sapiens | nuclear receptor subfamily 3 group C member 2 (NR3C2) |
| IGF1R | Homo sapiens | insulin like growth factor 1 receptor (IGF1R) |
| CCND1 | Homo sapiens | cyclin D1 (CCND1) |
| SCN9A | Homo sapiens | sodium voltage-gated channel alpha subunit 9 (SCN9A) |
| PALM | Homo sapiens | paralemmin (PALM) |
| ENPP2 | Homo sapiens | ectonucleotide pyrophosphatase/phosphodiesterase 2 (ENPP2) |
| ENPP1 | Homo sapiens | ectonucleotide pyrophosphatase/phosphodiesterase 1 (ENPP1) |
| PDGFRB | Homo sapiens | platelet derived growth factor receptor beta (PDGFRB) |
| G6PD | Homo sapiens | glucose-6-phosphate dehydrogenase (G6PD) |
| CCNE2 | Homo sapiens | cyclin E2 (CCNE2) |
| CCNE1 | Homo sapiens | cyclin E1 (CCNE1) |
| PLA2G10 | Homo sapiens | phospholipase A2 group X (PLA2G10) |
| PLAA | Homo sapiens | phospholipase A2 activating protein (PLAA) |
| NDUFB10 | Homo sapiens | NADH:ubiquinone oxidoreductase subunit B10 (NDUFB10) |
| NDUFB11 | Homo sapiens | NADH:ubiquinone oxidoreductase subunit B11 (NDUFB11) |
| PSEN2 | Homo sapiens | presenilin 2 (PSEN2) |
| SLC1A3 | Homo sapiens | solute carrier family 1 member 3 (SLC1A3) |
| CACNA1A | Homo sapiens | calcium voltage-gated channel subunit alpha1 A (CACNA1A) |
| PSEN1 | Homo sapiens | presenilin 1 (PSEN1) |
| MCHR1 | Homo sapiens | melanin concentrating hormone receptor 1 (MCHR1) |
| CACNA1G | Homo sapiens | calcium voltage-gated channel subunit alpha1 G (CACNA1G) |
| CCNB3 | Homo sapiens | cyclin B3 (CCNB3) |
| CACNA1I | Homo sapiens | calcium voltage-gated channel subunit alpha1 I (CACNA1I) |
| CCNB2 | Homo sapiens | cyclin B2 (CCNB2) |
| EBP | Homo sapiens | emopamil binding protein (sterol isomerase) (EBP) |
| CCNB1 | Homo sapiens | cyclin B1 (CCNB1) |
| GRK2 | Homo sapiens | G protein-coupled receptor kinase 2 (GRK2) |
| FUT7 | Homo sapiens | fucosyltransferase 7 (FUT7) |
| GRK5 | Homo sapiens | G protein-coupled receptor kinase 5 (GRK5) |
| NDUFV3 | Homo sapiens | NADH:ubiquinone oxidoreductase subunit V3 (NDUFV3) |
| NDUFV2 | Homo sapiens | NADH:ubiquinone oxidoreductase core subunit V2 (NDUFV2) |
| NDUFV1 | Homo sapiens | NADH:ubiquinone oxidoreductase core subunit V1 (NDUFV1) |
| FDPS | Homo sapiens | farnesyl diphosphate synthase (FDPS) |
| MGAM | Homo sapiens | maltase-glucoamylase (MGAM) |
| VCAM1 | Homo sapiens | vascular cell adhesion molecule 1 (VCAM1) |
| CAV2 | Homo sapiens | caveolin 2 (CAV2) |
| VDR | Homo sapiens | vitamin D (1,25- dihydroxyvitamin D3) receptor (VDR) |
| CAV1 | Homo sapiens | caveolin 1 (CAV1) |
| CLK4 | Homo sapiens | CDC like kinase 4 (CLK4) |
| GNAO1 | Homo sapiens | G protein subunit alpha o1 (GNAO1) |
| QDPR | Homo sapiens | quinoid dihydropteridine reductase (QDPR) |
| H1F0 | Homo sapiens | H1 histone family member 0 (H1F0) |
| IL6 | Homo sapiens | interleukin 6 (IL6) |
| IL5 | Homo sapiens | interleukin 5 (IL5) |
| CYP11B2 | Homo sapiens | cytochrome P450 family 11 subfamily B member 2 (CYP11B2) |
| CYP11B1 | Homo sapiens | cytochrome P450 family 11 subfamily B member 1 (CYP11B1) |
| GSK3B | Homo sapiens | glycogen synthase kinase 3 beta (GSK3B) |
| MTR | Homo sapiens | 5-methyltetrahydrofolate-homocysteine methyltransferase (MTR) |
| LTB4R | Homo sapiens | leukotriene B4 receptor (LTB4R) |
| CA1 | Homo sapiens | carbonic anhydrase 1 (CA1) |
| GRM5 | Homo sapiens | glutamate metabotropic receptor 5 (GRM5) |
| CYP26B1 | Homo sapiens | cytochrome P450 family 26 subfamily B member 1 (CYP26B1) |
| CA3 | Homo sapiens | carbonic anhydrase 3 (CA3) |
| CPT2 | Homo sapiens | carnitine palmitoyltransferase 2 (CPT2) |
| CA2 | Homo sapiens | carbonic anhydrase 2 (CA2) |
| CA4 | Homo sapiens | carbonic anhydrase 4 (CA4) |
| CA7 | Homo sapiens | carbonic anhydrase 7 (CA7) |
| CA6 | Homo sapiens | carbonic anhydrase 6 (CA6) |
| CA9 | Homo sapiens | carbonic anhydrase 9 (CA9) |
| SCN5A | Homo sapiens | sodium voltage-gated channel alpha subunit 5 (SCN5A) |
| YWHAG | Homo sapiens | tyrosine 3-monooxygenase/tryptophan 5-monooxygenase activation protein gamma (YWHAG) |
| MMP2 | Homo sapiens | matrix metallopeptidase 2 (MMP2) |
| PLA2G4B | Homo sapiens | phospholipase A2 group IVB (PLA2G4B) |
| MMP3 | Homo sapiens | matrix metallopeptidase 3 (MMP3) |
| PLA2G4A | Homo sapiens | phospholipase A2 group IVA (PLA2G4A) |
| IRAK4 | Homo sapiens | interleukin 1 receptor associated kinase 4 (IRAK4) |
| MMP8 | Homo sapiens | matrix metallopeptidase 8 (MMP8) |
| MMP9 | Homo sapiens | matrix metallopeptidase 9 (MMP9) |
| CCNA2 | Homo sapiens | cyclin A2 (CCNA2) |
| CCNA1 | Homo sapiens | cyclin A1 (CCNA1) |
| NDUFS8 | Homo sapiens | NADH:ubiquinone oxidoreductase core subunit S8 (NDUFS8) |
| CREB1 | Homo sapiens | cAMP responsive element binding protein 1 (CREB1) |
| NDUFS7 | Homo sapiens | NADH:ubiquinone oxidoreductase core subunit S7 (NDUFS7) |
| NDUFS6 | Homo sapiens | NADH:ubiquinone oxidoreductase subunit S6 (NDUFS6) |
| NDUFS5 | Homo sapiens | NADH:ubiquinone oxidoreductase subunit S5 (NDUFS5) |
| NDUFS4 | Homo sapiens | NADH:ubiquinone oxidoreductase subunit S4 (NDUFS4) |
| CRY2 | Homo sapiens | cryptochrome circadian clock 2 (CRY2) |
| NDUFS3 | Homo sapiens | NADH:ubiquinone oxidoreductase core subunit S3 (NDUFS3) |
| CRY1 | Homo sapiens | cryptochrome circadian clock 1 (CRY1) |
| NDUFS2 | Homo sapiens | NADH:ubiquinone oxidoreductase core subunit S2 (NDUFS2) |
| ATG4B | Homo sapiens | autophagy related 4B cysteine peptidase (ATG4B) |
| NDUFS1 | Homo sapiens | NADH:ubiquinone oxidoreductase core subunit S1 (NDUFS1) |
| PAOX | Homo sapiens | polyamine oxidase (PAOX) |
| PLA2G1B | Homo sapiens | phospholipase A2 group IB (PLA2G1B) |
| HMGCR | Homo sapiens | 3-hydroxy-3-methylglutaryl-CoA reductase (HMGCR) |
| AURKB | Homo sapiens | aurora kinase B (AURKB) |
| AURKA | Homo sapiens | aurora kinase A (AURKA) |
| INS | Homo sapiens | insulin (INS) |
| TNKS2 | Homo sapiens | tankyrase 2 (TNKS2) |
| CNR2 | Homo sapiens | cannabinoid receptor 2 (CNR2) |
| CBS | Homo sapiens | cystathionine-beta-synthase (CBS) |
| CNR1 | Homo sapiens | cannabinoid receptor 1 (CNR1) |
| MKNK1 | Homo sapiens | MAP kinase interacting serine/threonine kinase 1 (MKNK1) |
| MKNK2 | Homo sapiens | MAP kinase interacting serine/threonine kinase 2 (MKNK2) |
| PTK2B | Homo sapiens | protein tyrosine kinase 2 beta (PTK2B) |
| PLA2G2C | Homo sapiens | phospholipase A2 group IIC (PLA2G2C) |
| PLA2G2A | Homo sapiens | phospholipase A2 group IIA (PLA2G2A) |
| ATP2B4 | Homo sapiens | ATPase plasma membrane Ca2+ transporting 4 (ATP2B4) |
| MAPK14 | Homo sapiens | mitogen-activated protein kinase 14 (MAPK14) |
| MAPK12 | Homo sapiens | mitogen-activated protein kinase 12 (MAPK12) |
| MAPK13 | Homo sapiens | mitogen-activated protein kinase 13 (MAPK13) |
| MAPK10 | Homo sapiens | mitogen-activated protein kinase 10 (MAPK10) |
| MAPK11 | Homo sapiens | mitogen-activated protein kinase 11 (MAPK11) |
| CD4 | Homo sapiens | CD4 molecule (CD4) |
| CYP24A1 | Homo sapiens | cytochrome P450 family 24 subfamily A member 1 (CYP24A1) |
| ERCC4 | Homo sapiens | ERCC excision repair 4, endonuclease catalytic subunit (ERCC4) |
| GLB1 | Homo sapiens | galactosidase beta 1 (GLB1) |
| ERCC1 | Homo sapiens | ERCC excision repair 1, endonuclease non-catalytic subunit (ERCC1) |
| GLA | Homo sapiens | galactosidase alpha (GLA) |
| PNMT | Homo sapiens | phenylethanolamine N-methyltransferase (PNMT) |
| PRSS1 | Homo sapiens | protease, serine 1 (PRSS1) |
| RPL3 | Homo sapiens | ribosomal protein L3 (RPL3) |
| ABAT | Homo sapiens | 4-aminobutyrate aminotransferase (ABAT) |
| GPR174 | Homo sapiens | G protein-coupled receptor 174 (GPR174) |
| SCN11A | Homo sapiens | sodium voltage-gated channel alpha subunit 11 (SCN11A) |
| CYP1B1 | Homo sapiens | cytochrome P450 family 1 subfamily B member 1 (CYP1B1) |
| PRKCH | Homo sapiens | protein kinase C eta (PRKCH) |
| DAPK1 | Homo sapiens | death associated protein kinase 1 (DAPK1) |
| PRKCE | Homo sapiens | protein kinase C epsilon (PRKCE) |
| PRKCD | Homo sapiens | protein kinase C delta (PRKCD) |
| PRKCA | Homo sapiens | protein kinase C alpha (PRKCA) |
| TAAR1 | Homo sapiens | trace amine associated receptor 1 (TAAR1) |
| GABRG2 | Homo sapiens | gamma-aminobutyric acid type A receptor gamma2 subunit (GABRG2) |
| GANC | Homo sapiens | glucosidase alpha, neutral C (GANC) |
| SCN10A | Homo sapiens | sodium voltage-gated channel alpha subunit 10 (SCN10A) |
| MTAP | Homo sapiens | methylthioadenosine phosphorylase (MTAP) |
| PRKCQ | Homo sapiens | protein kinase C theta (PRKCQ) |
| ENPEP | Homo sapiens | glutamyl aminopeptidase (ENPEP) |
| PRKDC | Homo sapiens | protein kinase, DNA-activated, catalytic polypeptide (PRKDC) |
| ADH7 | Homo sapiens | alcohol dehydrogenase 7 (class IV), mu or sigma polypeptide (ADH7) |
| HIF1A | Homo sapiens | hypoxia inducible factor 1 alpha subunit (HIF1A) |
| NCSTN | Homo sapiens | nicastrin (NCSTN) |
| PPP3R1 | Homo sapiens | protein phosphatase 3 regulatory subunit B, alpha (PPP3R1) |
| TERT | Homo sapiens | telomerase reverse transcriptase (TERT) |
| ST3GAL1 | Homo sapiens | ST3 beta-galactoside alpha-2,3-sialyltransferase 1 (ST3GAL1) |
| FDFT1 | Homo sapiens | farnesyl-diphosphate farnesyltransferase 1 (FDFT1) |
| CHKA | Homo sapiens | choline kinase alpha (CHKA) |
| PLK1 | Homo sapiens | polo like kinase 1 (PLK1) |
| MTHFR | Homo sapiens | methylenetetrahydrofolate reductase (MTHFR) |
| CDC7 | Homo sapiens | cell division cycle 7 (CDC7) |
| SQLE | Homo sapiens | squalene epoxidase (SQLE) |
| GP9 | Homo sapiens | glycoprotein IX platelet (GP9) |
| SLC6A9 | Homo sapiens | solute carrier family 6 member 9 (SLC6A9) |
| PAH | Homo sapiens | phenylalanine hydroxylase (PAH) |
| CYP1A2 | Homo sapiens | cytochrome P450 family 1 subfamily A member 2 (CYP1A2) |
| CYP1A1 | Homo sapiens | cytochrome P450 family 1 subfamily A member 1 (CYP1A1) |
| CHRFAM7A | Homo sapiens | CHRNA7 (exons 5-10) and FAM7A (exons A-E) fusion (CHRFAM7A) |
| PAM | Homo sapiens | peptidylglycine alpha-amidating monooxygenase (PAM) |
| GABRB3 | Homo sapiens | gamma-aminobutyric acid type A receptor beta3 subunit (GABRB3) |
| GABRB2 | Homo sapiens | gamma-aminobutyric acid type A receptor beta2 subunit (GABRB2) |
| ITK | Homo sapiens | IL2 inducible T-cell kinase (ITK) |
| RARG | Homo sapiens | retinoic acid receptor gamma (RARG) |
| PYGL | Homo sapiens | phosphorylase, glycogen, liver (PYGL) |
| GLI1 | Homo sapiens | GLI family zinc finger 1 (GLI1) |
| SERPINA6 | Homo sapiens | serpin family A member 6 (SERPINA6) |
| SLC6A2 | Homo sapiens | solute carrier family 6 member 2 (SLC6A2) |
| SLC6A3 | Homo sapiens | solute carrier family 6 member 3 (SLC6A3) |
| SLC6A4 | Homo sapiens | solute carrier family 6 member 4 (SLC6A4) |
| PHF8 | Homo sapiens | PHD finger protein 8 (PHF8) |
| GPR139 | Homo sapiens | G protein-coupled receptor 139 (GPR139) |
| ANXA1 | Homo sapiens | annexin A1 (ANXA1) |
| TNNC1 | Homo sapiens | troponin C1, slow skeletal and cardiac type (TNNC1) |
| TACR2 | Homo sapiens | tachykinin receptor 2 (TACR2) |
| TACR1 | Homo sapiens | tachykinin receptor 1 (TACR1) |
| BACE1 | Homo sapiens | beta-secretase 1 (BACE1) |
| BACE2 | Homo sapiens | beta-site APP-cleaving enzyme 2 (BACE2) |
| TUBB2B | Homo sapiens | tubulin beta 2B class IIb (TUBB2B) |
| TUBB2A | Homo sapiens | tubulin beta 2A class IIa (TUBB2A) |
| RARA | Homo sapiens | retinoic acid receptor alpha (RARA) |
| RARB | Homo sapiens | retinoic acid receptor beta (RARB) |
| GART | Homo sapiens | phosphoribosylglycinamide formyltransferase, phosphoribosylglycinamide synthetase, phosphoribosylaminoimidazole synthetase (GART) |
| MGLL | Homo sapiens | monoglyceride lipase (MGLL) |
| NR1I3 | Homo sapiens | nuclear receptor subfamily 1 group I member 3 (NR1I3) |
| NR1I2 | Homo sapiens | nuclear receptor subfamily 1 group I member 2 (NR1I2) |
| NDUFA4L2 | Homo sapiens | NDUFA4, mitochondrial complex associated like 2 (NDUFA4L2) |
| ADCY1 | Homo sapiens | adenylate cyclase 1 (ADCY1) |
| ASCL1 | Homo sapiens | achaete-scute family bHLH transcription factor 1 (ASCL1) |
| ADCY5 | Homo sapiens | adenylate cyclase 5 (ADCY5) |
| CHRND | Homo sapiens | cholinergic receptor nicotinic delta subunit (CHRND) |
| CHRNG | Homo sapiens | cholinergic receptor nicotinic gamma subunit (CHRNG) |
| ALOX5 | Homo sapiens | arachidonate 5-lipoxygenase (ALOX5) |
| CHRNE | Homo sapiens | cholinergic receptor nicotinic epsilon subunit (CHRNE) |
| USP1 | Homo sapiens | ubiquitin specific peptidase 1 (USP1) |
| BRD4 | Homo sapiens | bromodomain containing 4 (BRD4) |
| GABRA2 | Homo sapiens | gamma-aminobutyric acid type A receptor alpha2 subunit (GABRA2) |
| BCHE | Homo sapiens | butyrylcholinesterase (BCHE) |
| F10 | Homo sapiens | coagulation factor X (F10) |
| GABRA5 | Homo sapiens | gamma-aminobutyric acid type A receptor alpha5 subunit (GABRA5) |
| AGL | Homo sapiens | amylo-alpha-1, 6-glucosidase, 4-alpha-glucanotransferase (AGL) |
| NR1H2 | Homo sapiens | nuclear receptor subfamily 1 group H member 2 (NR1H2) |
| F11 | Homo sapiens | coagulation factor XI (F11) |
| GSR | Homo sapiens | glutathione-disulfide reductase (GSR) |
| NR1H4 | Homo sapiens | nuclear receptor subfamily 1 group H member 4 (NR1H4) |
| NR1H3 | Homo sapiens | nuclear receptor subfamily 1 group H member 3 (NR1H3) |
| GPR4 | Homo sapiens | G protein-coupled receptor 4 (GPR4) |
| TUBB4B | Homo sapiens | tubulin beta 4B class IVb (TUBB4B) |
| TUBB4A | Homo sapiens | tubulin beta 4A class IVa (TUBB4A) |
| KLKB1 | Homo sapiens | kallikrein B1 (KLKB1) |
| HSPA1A | Homo sapiens | heat shock protein family A (Hsp70) member 1A (HSPA1A) |
| CHRM2 | Homo sapiens | cholinergic receptor muscarinic 2 (CHRM2) |
| RAB3B | Homo sapiens | RAB3B, member RAS oncogene family (RAB3B) |
| CHRM3 | Homo sapiens | cholinergic receptor muscarinic 3 (CHRM3) |
| MTFMT | Homo sapiens | mitochondrial methionyl-tRNA formyltransferase (MTFMT) |
| CHRM1 | Homo sapiens | cholinergic receptor muscarinic 1 (CHRM1) |
| CHRM4 | Homo sapiens | cholinergic receptor muscarinic 4 (CHRM4) |
| CHRM5 | Homo sapiens | cholinergic receptor muscarinic 5 (CHRM5) |
| AHR | Homo sapiens | aryl hydrocarbon receptor (AHR) |
| FGF2 | Homo sapiens | fibroblast growth factor 2 (FGF2) |
| TNF | Homo sapiens | tumor necrosis factor (TNF) |
| TUBB8 | Homo sapiens | tubulin beta 8 class VIII (TUBB8) |
| CYSLTR1 | Homo sapiens | cysteinyl leukotriene receptor 1 (CYSLTR1) |
| TUBB6 | Homo sapiens | tubulin beta 6 class V (TUBB6) |
| IGHG1 | Homo sapiens | immunoglobulin heavy constant gamma 1 (G1m marker) (IGHG1) |
| TUBB3 | Homo sapiens | tubulin beta 3 class III (TUBB3) |
| TUBB1 | Homo sapiens | tubulin beta 1 class VI (TUBB1) |
| MEN1 | Homo sapiens | menin 1 (MEN1) |
| MAP2K4 | Homo sapiens | mitogen-activated protein kinase kinase 4 (MAP2K4) |
| MAP2K1 | Homo sapiens | mitogen-activated protein kinase kinase 1 (MAP2K1) |
| IGFBP5 | Homo sapiens | insulin like growth factor binding protein 5 (IGFBP5) |
| IGFBP3 | Homo sapiens | insulin like growth factor binding protein 3 (IGFBP3) |
| MTNR1A | Homo sapiens | melatonin receptor 1A (MTNR1A) |
| MTNR1B | Homo sapiens | melatonin receptor 1B (MTNR1B) |
| SLCO2A1 | Homo sapiens | solute carrier organic anion transporter family member 2A1 (SLCO2A1) |
| PGR | Homo sapiens | progesterone receptor (PGR) |
| FKBP5 | Homo sapiens | FK506 binding protein 5 (FKBP5) |
| CSF1R | Homo sapiens | colony stimulating factor 1 receptor (CSF1R) |
| TNKS | Homo sapiens | tankyrase (TNKS) |
| GLO1 | Homo sapiens | glyoxalase I (GLO1) |
| TBXA2R | Homo sapiens | thromboxane A2 receptor (TBXA2R) |
| ABL1 | Homo sapiens | ABL proto-oncogene 1, non-receptor tyrosine kinase (ABL1) |
| DRD1 | Homo sapiens | dopamine receptor D1 (DRD1) |
| MAP2K7 | Homo sapiens | mitogen-activated protein kinase kinase 7 (MAP2K7) |
| DRD2 | Homo sapiens | dopamine receptor D2 (DRD2) |
| DRD3 | Homo sapiens | dopamine receptor D3 (DRD3) |
| DRD4 | Homo sapiens | dopamine receptor D4 (DRD4) |
| MCL1 | Homo sapiens | BCL2 family apoptosis regulator (MCL1) |
| DRD5 | Homo sapiens | dopamine receptor D5 (DRD5) |
| ESRRA | Homo sapiens | estrogen related receptor alpha (ESRRA) |
| ESRRG | Homo sapiens | estrogen related receptor gamma (ESRRG) |
| DHCR24 | Homo sapiens | 24-dehydrocholesterol reductase (DHCR24) |
| CDK9 | Homo sapiens | cyclin dependent kinase 9 (CDK9) |
| ADCY10 | Homo sapiens | adenylate cyclase 10, soluble (ADCY10) |
| CDK8 | Homo sapiens | cyclin dependent kinase 8 (CDK8) |
| CDK6 | Homo sapiens | cyclin dependent kinase 6 (CDK6) |
| P4HA1 | Homo sapiens | prolyl 4-hydroxylase subunit alpha 1 (P4HA1) |
| CDK5 | Homo sapiens | cyclin dependent kinase 5 (CDK5) |
| CDK4 | Homo sapiens | cyclin dependent kinase 4 (CDK4) |
| ALB | Homo sapiens | albumin (ALB) |
| CDK2 | Homo sapiens | cyclin dependent kinase 2 (CDK2) |
| BCL2 | Homo sapiens | BCL2, apoptosis regulator (BCL2) |
| CDK1 | Homo sapiens | cyclin dependent kinase 1 (CDK1) |
| IARS | Homo sapiens | isoleucyl-tRNA synthetase (IARS) |
| DHCR7 | Homo sapiens | 7-dehydrocholesterol reductase (DHCR7) |
| MAP3K14 | Homo sapiens | mitogen-activated protein kinase kinase kinase 14 (MAP3K14) |
| BCL2L1 | Homo sapiens | BCL2 like 1 (BCL2L1) |
| TUBA8 | Homo sapiens | tubulin alpha 8 (TUBA8) |
| MAP3K12 | Homo sapiens | mitogen-activated protein kinase kinase kinase 12 (MAP3K12) |
| ALK | Homo sapiens | anaplastic lymphoma receptor tyrosine kinase (ALK) |
| DAGLA | Homo sapiens | diacylglycerol lipase alpha (DAGLA) |
| CALCOCO2 | Homo sapiens | calcium binding and coiled-coil domain 2 (CALCOCO2) |
| AMD1 | Homo sapiens | adenosylmethionine decarboxylase 1 (AMD1) |
| ADRA1D | Homo sapiens | adrenoceptor alpha 1D (ADRA1D) |
| ADRA1B | Homo sapiens | adrenoceptor alpha 1B (ADRA1B) |
| ADRA1A | Homo sapiens | adrenoceptor alpha 1A (ADRA1A) |
| FTCD | Homo sapiens | formimidoyltransferase cyclodeaminase (FTCD) |
| ADAMTS5 | Homo sapiens | ADAM metallopeptidase with thrombospondin type 1 motif 5 (ADAMTS5) |
| GGPS1 | Homo sapiens | geranylgeranyl diphosphate synthase 1 (GGPS1) |
| AVPR2 | Homo sapiens | arginine vasopressin receptor 2 (AVPR2) |
| AMT | Homo sapiens | aminomethyltransferase (AMT) |
| SORD | Homo sapiens | sorbitol dehydrogenase (SORD) |
| ADRA2C | Homo sapiens | adrenoceptor alpha 2C (ADRA2C) |
| ADRA2B | Homo sapiens | adrenoceptor alpha 2B (ADRA2B) |
| ADRA2A | Homo sapiens | adrenoceptor alpha 2A (ADRA2A) |
| PKM | Homo sapiens | pyruvate kinase, muscle (PKM) |
| LCK | Homo sapiens | LCK proto-oncogene, Src family tyrosine kinase (LCK) |
| LCT | Homo sapiens | lactase (LCT) |
| HDAC2 | Homo sapiens | histone deacetylase 2 (HDAC2) |
| HDAC3 | Homo sapiens | histone deacetylase 3 (HDAC3) |
| HDAC1 | Homo sapiens | histone deacetylase 1 (HDAC1) |
| NOD1 | Homo sapiens | nucleotide binding oligomerization domain containing 1 (NOD1) |
| TTK | Homo sapiens | TTK protein kinase (TTK) |
| PLG | Homo sapiens | plasminogen (PLG) |
| PDHB | Homo sapiens | pyruvate dehydrogenase (lipoamide) beta (PDHB) |
| CYP3A4 | Homo sapiens | cytochrome P450 family 3 subfamily A member 4 (CYP3A4) |
| CYP3A5 | Homo sapiens | cytochrome P450 family 3 subfamily A member 5 (CYP3A5) |
| CYP3A7 | Homo sapiens | cytochrome P450 family 3 subfamily A member 7 (CYP3A7) |
| TTR | Homo sapiens | transthyretin (TTR) |
| RBBP9 | Homo sapiens | RB binding protein 9, serine hydrolase (RBBP9) |
| TNNI3 | Homo sapiens | troponin I3, cardiac type (TNNI3) |
| RCVRN | Homo sapiens | recoverin (RCVRN) |
| KCNJ10 | Homo sapiens | potassium voltage-gated channel subfamily J member 10 (KCNJ10) |
| TRPA1 | Homo sapiens | transient receptor potential cation channel subfamily A member 1 (TRPA1) |
| KCNJ11 | Homo sapiens | potassium voltage-gated channel subfamily J member 11 (KCNJ11) |
| CMA1 | Homo sapiens | chymase 1 (CMA1) |
| KCNJ12 | Homo sapiens | potassium voltage-gated channel subfamily J member 12 (KCNJ12) |
| CYP51A1 | Homo sapiens | cytochrome P450 family 51 subfamily A member 1 (CYP51A1) |
| KCNJ14 | Homo sapiens | potassium voltage-gated channel subfamily J member 14 (KCNJ14) |
| KCNJ15 | Homo sapiens | potassium voltage-gated channel subfamily J member 15 (KCNJ15) |
| PTPN11 | Homo sapiens | protein tyrosine phosphatase, non-receptor type 11 (PTPN11) |
| MTOR | Homo sapiens | mechanistic target of rapamycin (MTOR) |
| P2RX7 | Homo sapiens | purinergic receptor P2X 7 (P2RX7) |
| IMPDH2 | Homo sapiens | inosine monophosphate dehydrogenase 2 (IMPDH2) |
| FOLR3 | Homo sapiens | folate receptor 3 (FOLR3) |
| FOLR2 | Homo sapiens | folate receptor beta (FOLR2) |
| METAP2 | Homo sapiens | methionyl aminopeptidase 2 (METAP2) |
| APP | Homo sapiens | amyloid beta precursor protein (APP) |
| FAAH | Homo sapiens | fatty acid amide hydrolase (FAAH) |
| GBA2 | Homo sapiens | glucosylceramidase beta 2 (GBA2) |
| EDNRA | Homo sapiens | endothelin receptor type A (EDNRA) |
| PNP | Homo sapiens | purine nucleoside phosphorylase (PNP) |
| RPS6KA5 | Homo sapiens | ribosomal protein S6 kinase A5 (RPS6KA5) |
| KAT5 | Homo sapiens | lysine acetyltransferase 5 (KAT5) |
| CHEK2 | Homo sapiens | checkpoint kinase 2 (CHEK2) |
| CHEK1 | Homo sapiens | checkpoint kinase 1 (CHEK1) |
| GLUL | Homo sapiens | glutamate-ammonia ligase (GLUL) |
| PTGDR | Homo sapiens | prostaglandin D2 receptor (PTGDR) |
| UTS2R | Homo sapiens | urotensin 2 receptor (UTS2R) |
| CPT1A | Homo sapiens | carnitine palmitoyltransferase 1A (CPT1A) |
| ARRDC3 | Homo sapiens | arrestin domain containing 3 (ARRDC3) |
| P3H2 | Homo sapiens | prolyl 3-hydroxylase 2 (P3H2) |
| P3H1 | Homo sapiens | prolyl 3-hydroxylase 1 (P3H1) |
| SLC6A11 | Homo sapiens | solute carrier family 6 member 11 (SLC6A11) |
| P3H3 | Homo sapiens | prolyl 3-hydroxylase 3 (P3H3) |
| EZR | Homo sapiens | ezrin (EZR) |
| PTGES | Homo sapiens | prostaglandin E synthase (PTGES) |
| SLC22A3 | Homo sapiens | solute carrier family 22 member 3 (SLC22A3) |
| PTGFR | Homo sapiens | prostaglandin F receptor (PTGFR) |
| SLC22A6 | Homo sapiens | solute carrier family 22 member 6 (SLC22A6) |
| MAOB | Homo sapiens | monoamine oxidase B (MAOB) |
| PDE1C | Homo sapiens | phosphodiesterase 1C (PDE1C) |
| MAOA | Homo sapiens | monoamine oxidase A (MAOA) |
| SLC22A1 | Homo sapiens | solute carrier family 22 member 1 (SLC22A1) |
| CRHR1 | Homo sapiens | corticotropin releasing hormone receptor 1 (CRHR1) |
| UGCG | Homo sapiens | UDP-glucose ceramide glucosyltransferase (UGCG) |
| CYP27B1 | Homo sapiens | cytochrome P450 family 27 subfamily B member 1 (CYP27B1) |
| NUAK1 | Homo sapiens | NUAK family kinase 1 (NUAK1) |
| MAP2 | Homo sapiens | microtubule associated protein 2 (MAP2) |
| NAALAD2 | Homo sapiens | N-acetylated alpha-linked acidic dipeptidase 2 (NAALAD2) |
| MAP4 | Homo sapiens | microtubule associated protein 4 (MAP4) |
| TRPM8 | Homo sapiens | transient receptor potential cation channel subfamily M member 8 (TRPM8) |
| NTSR1 | Homo sapiens | neurotensin receptor 1 (NTSR1) |
| INSR | Homo sapiens | insulin receptor (INSR) |
| PDE2A | Homo sapiens | phosphodiesterase 2A (PDE2A) |
| TYR | Homo sapiens | tyrosinase (TYR) |
| PARP10 | Homo sapiens | poly (ADP-ribose) polymerase family member 10 (PARP10) |
| AKR1B10 | Homo sapiens | aldo-keto reductase family 1 member B10 (AKR1B10) |
| PDE3A | Homo sapiens | phosphodiesterase 3A (PDE3A) |
| SLC22A8 | Homo sapiens | solute carrier family 22 member 8 (SLC22A8) |
| TOP2A | Homo sapiens | topoisomerase (DNA) II alpha (TOP2A) |
| BRS3 | Homo sapiens | bombesin receptor subtype 3 (BRS3) |
| SLC22A12 | Homo sapiens | solute carrier family 22 member 12 (SLC22A12) |
| GABRR1 | Homo sapiens | gamma-aminobutyric acid type A receptor rho1 subunit (GABRR1) |
| FFAR4 | Homo sapiens | free fatty acid receptor 4 (FFAR4) |
| FFAR1 | Homo sapiens | free fatty acid receptor 1 (FFAR1) |
| PSENEN | Homo sapiens | presenilin enhancer gamma-secretase subunit (PSENEN) |
| PRMT6 | Homo sapiens | protein arginine methyltransferase 6 (PRMT6) |
| PRMT8 | Homo sapiens | protein arginine methyltransferase 8 (PRMT8) |
| PARP3 | Homo sapiens | poly (ADP-ribose) polymerase family member 3 (PARP3) |
| PARP1 | Homo sapiens | poly (ADP-ribose) polymerase 1 (PARP1) |
| PRMT1 | Homo sapiens | protein arginine methyltransferase 1 (PRMT1) |
| PARP2 | Homo sapiens | poly (ADP-ribose) polymerase 2 (PARP2) |
| TUBB | Homo sapiens | tubulin beta class I (TUBB) |
| GPBAR1 | Homo sapiens | G protein-coupled bile acid receptor 1 (GPBAR1) |
| TUBG2 | Homo sapiens | tubulin gamma 2 (TUBG2) |
| AVPR1A | Homo sapiens | arginine vasopressin receptor 1A (AVPR1A) |
| TYK2 | Homo sapiens | tyrosine kinase 2 (TYK2) |
| TUBG1 | Homo sapiens | tubulin gamma 1 (TUBG1) |
| HCK | Homo sapiens | HCK proto-oncogene, Src family tyrosine kinase (HCK) |
| ADRB3 | Homo sapiens | adrenoceptor beta 3 (ADRB3) |
| CCKBR | Homo sapiens | cholecystokinin B receptor (CCKBR) |
| TNNT2 | Homo sapiens | troponin T2, cardiac type (TNNT2) |
| MAPT | Homo sapiens | microtubule associated protein tau (MAPT) |
| TLR4 | Homo sapiens | toll like receptor 4 (TLR4) |
| TLR2 | Homo sapiens | toll like receptor 2 (TLR2) |
| RAB7A | Homo sapiens | RAB7A, member RAS oncogene family (RAB7A) |
| OPRD1 | Homo sapiens | opioid receptor delta 1 (OPRD1) |
| PTGER4 | Homo sapiens | prostaglandin E receptor 4 (PTGER4) |
| ADH1C | Homo sapiens | alcohol dehydrogenase 1C (class I), gamma polypeptide (ADH1C) |
| ADH1B | Homo sapiens | alcohol dehydrogenase 1B (class I), beta polypeptide (ADH1B) |
| PTGER1 | Homo sapiens | prostaglandin E receptor 1 (PTGER1) |
| ADH1A | Homo sapiens | alcohol dehydrogenase 1A (class I), alpha polypeptide (ADH1A) |
| CYP3A51P | Homo sapiens | cytochrome P450 family 3 subfamily A member 51, pseudogene (CYP3A51P) |
| PTGER2 | Homo sapiens | prostaglandin E receptor 2 (PTGER2) |
| PTGER3 | Homo sapiens | prostaglandin E receptor 3 (PTGER3) |
| TUBD1 | Homo sapiens | tubulin delta 1 (TUBD1) |
| TYMS | Homo sapiens | thymidylate synthetase (TYMS) |
| TYMP | Homo sapiens | thymidine phosphorylase (TYMP) |
| PPP2CA | Homo sapiens | protein phosphatase 2 catalytic subunit alpha (PPP2CA) |
| CXCR1 | Homo sapiens | C-X-C motif chemokine receptor 1 (CXCR1) |
| MAP4K4 | Homo sapiens | mitogen-activated protein kinase kinase kinase kinase 4 (MAP4K4) |
| GABRQ | Homo sapiens | gamma-aminobutyric acid type A receptor theta subunit (GABRQ) |
| CA12 | Homo sapiens | carbonic anhydrase 12 (CA12) |
| NQO1 | Homo sapiens | NAD (P)H quinone dehydrogenase 1 (NQO1) |
| NQO2 | Homo sapiens | NAD (P)H quinone dehydrogenase 2 (NQO2) |
| PTGES2 | Homo sapiens | prostaglandin E synthase 2 (PTGES2) |
| TUBE1 | Homo sapiens | tubulin epsilon 1 (TUBE1) |
| FAP | Homo sapiens | fibroblast activation protein alpha (FAP) |
| AXL | Homo sapiens | AXL receptor tyrosine kinase (AXL) |
| GSTA1 | Homo sapiens | glutathione S-transferase alpha 1 (GSTA1) |
| CDK5R1 | Homo sapiens | cyclin dependent kinase 5 regulatory subunit 1 (CDK5R1) |
| NDUFA13 | Homo sapiens | NADH:ubiquinone oxidoreductase subunit A13 (NDUFA13) |
| KDM1A | Homo sapiens | lysine demethylase 1A (KDM1A) |
| NDUFA11 | Homo sapiens | NADH:ubiquinone oxidoreductase subunit A11 (NDUFA11) |
| NDUFA12 | Homo sapiens | NADH:ubiquinone oxidoreductase subunit A12 (NDUFA12) |
| NDUFA10 | Homo sapiens | NADH:ubiquinone oxidoreductase subunit A10 (NDUFA10) |
| PVR | Homo sapiens | poliovirus receptor (PVR) |
| PDE8B | Homo sapiens | phosphodiesterase 8B (PDE8B) |
| CA14 | Homo sapiens | carbonic anhydrase 14 (CA14) |
| CA13 | Homo sapiens | carbonic anhydrase 13 (CA13) |
| CHRNB2 | Homo sapiens | cholinergic receptor nicotinic beta 2 subunit (CHRNB2) |
| CHRNB1 | Homo sapiens | cholinergic receptor nicotinic beta 1 subunit (CHRNB1) |
| CHRNB4 | Homo sapiens | cholinergic receptor nicotinic beta 4 subunit (CHRNB4) |
| PHYH | Homo sapiens | phytanoyl-CoA 2-hydroxylase (PHYH) |
| KDM2A | Homo sapiens | lysine demethylase 2A (KDM2A) |
| CHRNB3 | Homo sapiens | cholinergic receptor nicotinic beta 3 subunit (CHRNB3) |
| APLP1 | Homo sapiens | amyloid beta precursor like protein 1 (APLP1) |
| NPY1R | Homo sapiens | neuropeptide Y receptor Y1 (NPY1R) |
| MPEG1 | Homo sapiens | macrophage expressed 1 (MPEG1) |
| FOLH1 | Homo sapiens | folate hydrolase 1 (FOLH1) |
| PDE9A | Homo sapiens | phosphodiesterase 9A (PDE9A) |
| ABCG2 | Homo sapiens | ATP binding cassette subfamily G member 2 (Junior blood group) (ABCG2) |
| CHRNA1 | Homo sapiens | cholinergic receptor nicotinic alpha 1 subunit (CHRNA1) |
| CHRNA3 | Homo sapiens | cholinergic receptor nicotinic alpha 3 subunit (CHRNA3) |
| CHRNA2 | Homo sapiens | cholinergic receptor nicotinic alpha 2 subunit (CHRNA2) |
| CHRNA5 | Homo sapiens | cholinergic receptor nicotinic alpha 5 subunit (CHRNA5) |
| CHRNA4 | Homo sapiens | cholinergic receptor nicotinic alpha 4 subunit (CHRNA4) |
| CHRNA7 | Homo sapiens | cholinergic receptor nicotinic alpha 7 subunit (CHRNA7) |
| ALOX15 | Homo sapiens | arachidonate 15-lipoxygenase (ALOX15) |
| CHRNA6 | Homo sapiens | cholinergic receptor nicotinic alpha 6 subunit (CHRNA6) |
| CHRNA9 | Homo sapiens | cholinergic receptor nicotinic alpha 9 subunit (CHRNA9) |
| ADRB1 | Homo sapiens | adrenoceptor beta 1 (ADRB1) |
| ALOX12 | Homo sapiens | arachidonate 12-lipoxygenase, 12S type (ALOX12) |
| ADRB2 | Homo sapiens | adrenoceptor beta 2 (ADRB2) |
| DPP4 | Homo sapiens | dipeptidyl peptidase 4 (DPP4) |
| HRH1 | Homo sapiens | histamine receptor H1 (HRH1) |
| DPP7 | Homo sapiens | dipeptidyl peptidase 7 (DPP7) |
| DPP8 | Homo sapiens | dipeptidyl peptidase 8 (DPP8) |
| MAN2A1 | Homo sapiens | mannosidase alpha class 2A member 1 (MAN2A1) |
| DPP9 | Homo sapiens | dipeptidyl peptidase 9 (DPP9) |
| NPC1L1 | Homo sapiens | NPC1 like intracellular cholesterol transporter 1 (NPC1L1) |
| HRH2 | Homo sapiens | histamine receptor H2 (HRH2) |
| HRH4 | Homo sapiens | histamine receptor H4 (HRH4) |
| EPHX2 | Homo sapiens | epoxide hydrolase 2 (EPHX2) |
| EPHX1 | Homo sapiens | epoxide hydrolase 1 (EPHX1) |
| TRPV3 | Homo sapiens | transient receptor potential cation channel subfamily V member 3 (TRPV3) |
| TRPV1 | Homo sapiens | transient receptor potential cation channel subfamily V member 1 (TRPV1) |
| ESR1 | Homo sapiens | estrogen receptor 1 (ESR1) |
| SELE | Homo sapiens | selectin E (SELE) |
| ESR2 | Homo sapiens | estrogen receptor 2 (ESR2) |
| SELP | Homo sapiens | selectin P (SELP) |
| NR4A1 | Homo sapiens | nuclear receptor subfamily 4 group A member 1 (NR4A1) |
| SELL | Homo sapiens | selectin L (SELL) |
| NMUR2 | Homo sapiens | neuromedin U receptor 2 (NMUR2) |
| MAN2B1 | Homo sapiens | mannosidase alpha class 2B member 1 (MAN2B1) |
| PI4KB | Homo sapiens | phosphatidylinositol 4-kinase beta (PI4KB) |
| KDM5A | Homo sapiens | lysine demethylase 5A (KDM5A) |
| UCN | Homo sapiens | urocortin (UCN) |
| KDM5C | Homo sapiens | lysine demethylase 5C (KDM5C) |
| KDM5D | Homo sapiens | lysine demethylase 5D (KDM5D) |
| THRB | Homo sapiens | thyroid hormone receptor beta (THRB) |
| THRA | Homo sapiens | thyroid hormone receptor, alpha (THRA) |
| MLNR | Homo sapiens | motilin receptor (MLNR) |
| PDE3B | Homo sapiens | phosphodiesterase 3B (PDE3B) |
| DBH | Homo sapiens | dopamine beta-hydroxylase (DBH) |
| POLB | Homo sapiens | DNA polymerase beta (POLB) |
| CA5B | Homo sapiens | carbonic anhydrase 5B (CA5B) |
| CA5A | Homo sapiens | carbonic anhydrase 5A (CA5A) |
| CASP3 | Homo sapiens | caspase 3 (CASP3) |
| PDE4B | Homo sapiens | phosphodiesterase 4B (PDE4B) |
| BDKRB1 | Homo sapiens | bradykinin receptor B1 (BDKRB1) |
| PDE4A | Homo sapiens | phosphodiesterase 4A (PDE4A) |
| POLL | Homo sapiens | DNA polymerase lambda (POLL) |
| POLK | Homo sapiens | DNA polymerase kappa (POLK) |
| NOS1 | Homo sapiens | nitric oxide synthase 1 (NOS1) |
| POLH | Homo sapiens | DNA polymerase eta (POLH) |
| ABCC4 | Homo sapiens | ATP binding cassette subfamily C member 4 (ABCC4) |
| ABCC1 | Homo sapiens | ATP binding cassette subfamily C member 1 (ABCC1) |
| PTGIR | Homo sapiens | prostaglandin I2 (prostacyclin) receptor (IP) (PTGIR) |
| NPY5R | Homo sapiens | neuropeptide Y receptor Y5 (NPY5R) |
| ABCC8 | Homo sapiens | ATP binding cassette subfamily C member 8 (ABCC8) |
| PDE4D | Homo sapiens | phosphodiesterase 4D (PDE4D) |
| PDE4C | Homo sapiens | phosphodiesterase 4C (PDE4C) |
| ABCC9 | Homo sapiens | ATP binding cassette subfamily C member 9 (ABCC9) |
| OPRM1 | Homo sapiens | opioid receptor mu 1 (OPRM1) |
| TGFBR1 | Homo sapiens | transforming growth factor beta receptor 1 (TGFBR1) |
| DNM1 | Homo sapiens | dynamin 1 (DNM1) |
| EHHADH | Homo sapiens | enoyl-CoA hydratase and 3-hydroxyacyl CoA dehydrogenase (EHHADH) |
| PDE5A | Homo sapiens | phosphodiesterase 5A (PDE5A) |
| KDM7A | Homo sapiens | lysine demethylase 7A (KDM7A) |
| CAMK2B | Homo sapiens | calcium/calmodulin dependent protein kinase II beta (CAMK2B) |
| HCN4 | Homo sapiens | hyperpolarization activated cyclic nucleotide gated potassium channel 4 (HCN4) |
| DDC | Homo sapiens | dopa decarboxylase (DDC) |
| HPGD | Homo sapiens | hydroxyprostaglandin dehydrogenase 15- (NAD) (HPGD) |
| OPRL1 | Homo sapiens | opioid related nociceptin receptor 1 (OPRL1) |
| ATP1A1 | Homo sapiens | ATPase Na+/K+ transporting subunit alpha 1 (ATP1A1) |
| MAPK9 | Homo sapiens | mitogen-activated protein kinase 9 (MAPK9) |
| OGFRL1 | Homo sapiens | opioid growth factor receptor like 1 (OGFRL1) |
| MAPK8 | Homo sapiens | mitogen-activated protein kinase 8 (MAPK8) |
| PDE11A | Homo sapiens | phosphodiesterase 11A (PDE11A) |
| MUC2 | Homo sapiens | mucin 2, oligomeric mucus/gel-forming (MUC2) |
| ERBB2 | Homo sapiens | erb-b2 receptor tyrosine kinase 2 (ERBB2) |
| S1PR1 | Homo sapiens | sphingosine-1-phosphate receptor 1 (S1PR1) |
| MAPK1 | Homo sapiens | mitogen-activated protein kinase 1 (MAPK1) |
| S1PR3 | Homo sapiens | sphingosine-1-phosphate receptor 3 (S1PR3) |
| S1PR2 | Homo sapiens | sphingosine-1-phosphate receptor 2 (S1PR2) |
| S1PR5 | Homo sapiens | sphingosine-1-phosphate receptor 5 (S1PR5) |
| S1PR4 | Homo sapiens | sphingosine-1-phosphate receptor 4 (S1PR4) |
| SNCA | Homo sapiens | synuclein alpha (SNCA) |
| MAPK3 | Homo sapiens | mitogen-activated protein kinase 3 (MAPK3) |
| KDM4A | Homo sapiens | lysine demethylase 4A (KDM4A) |
| KDM4C | Homo sapiens | lysine demethylase 4C (KDM4C) |
| NOS2 | Homo sapiens | nitric oxide synthase 2 (NOS2) |
| NOS3 | Homo sapiens | nitric oxide synthase 3 (NOS3) |
| POLM | Homo sapiens | DNA polymerase mu (POLM) |
| KDM4E | Homo sapiens | lysine demethylase 4E (KDM4E) |
| LIMK2 | Homo sapiens | LIM domain kinase 2 (LIMK2) |
| OPRK1 | Homo sapiens | opioid receptor kappa 1 (OPRK1) |
| PDE10A | Homo sapiens | phosphodiesterase 10A (PDE10A) |
| RPS6KB1 | Homo sapiens | ribosomal protein S6 kinase B1 (RPS6KB1) |
| XPNPEP1 | Homo sapiens | X-prolyl aminopeptidase 1 (XPNPEP1) |
| SCD | Homo sapiens | stearoyl-CoA desaturase (SCD) |
| XPNPEP2 | Homo sapiens | X-prolyl aminopeptidase 2 (XPNPEP2) |
| ALOX5AP | Homo sapiens | arachidonate 5-lipoxygenase activating protein (ALOX5AP) |
| PDE7A | Homo sapiens | phosphodiesterase 7A (PDE7A) |
| HCN1 | Homo sapiens | hyperpolarization activated cyclic nucleotide gated potassium channel 1 (HCN1) |
